# Supplementary material for: Cohort profile: a prospective Australian cohort study of women’s reproductive characteristics and risk of chronic disease from menarche to premenopause (M-PreM)
Source: BMJ Open. 2022 Oct 28;12(10):e064333. doi: 10.1136/bmjopen-2022-064333 (PMC9621184; doi:10.1136/bmjopen-2022-064333)
Supplement: Author's manuscript [file bmjopen-2022-064333.draft_revisions.pdf]

BMJ Open is committed to open peer review. As part of this commitment we make the peer review history of every article we publish publicly available.

When an article is published we post the peer reviewers' comments and the authors' responses online. We also post the versions of the paper that were used during peer review. These are the versions that the peer review comments apply to.

The versions of the paper that follow are the versions that were submitted during the peer review process. They are not the versions of record or the final published versions. They should not be cited or distributed as the published version of this manuscript.

BMJ Open is an open access journal and the full, final, typeset and author-corrected version of record of the manuscript is available on our site with no access controls, subscription charges or pay-per-view fees (http://bmjopen.bmj.com).

If you have any questions on BMJ Open's open peer review process please email info.bmjopen@bmj.com

BMJ Open

Cohort profile: A prospective Australian cohort study of women's reproductive characteristics and risk of chronic disease from menarche to premenopause (M-PreM)

Journal:	*BMJ Open*
Manuscript ID	bmjopen-2022-064333
Article Type:	Cohort profile
Date Submitted by the Author:	28-Apr-2022
Complete List of Authors:	Chan, Hsiu-Wen; The University of Queensland, School of Public Health<br>Dharmage, Shyamali; The University of Melbourne, School of Population and Global Health<br>Dobson, A; The University of Queensland, School of Public Health<br>Chung, Hsing-Fang; The University of Queensland, School of Public Health<br>Loxton, Deborah; The University of Newcastle, Centre for Women's Health Research<br>Doust, Jenny; The University of Queensland, School of Public Health<br>Montgomery, Grant ; The University of Queensland Institute for Molecular Bioscience<br>Stamatakis, Emmanuel; The University of Sydney, Charles Perkins Centre<br>Huxley, Rachel; Deakin University, Faculty of Health; University of New South Wales, The George Institute for Global Health<br>Hamer, Mark; University College London, Epidemiology and public health<br>Abbott, Jason ; University of New South Wales, School of Women's and Children's Health; Royal Hospital for Women, Gynaecological Research and Clinical Evaluation (GRACE) Unit<br>Yeap, Bu; The University of Western Australia, Medical School; Fiona Stanley Hospital, Department of Endocrinology and Diabetes<br>Visser, Jenny; Erasmus MC, University Medical Center<br>McIntyre, Harold; The University of Queensland, School of Medicine; Mater Misericordiae Health Services Brisbane Ltd<br>Mielke , Gregore ; The University of Queensland, School of Public Health<br>Mishra, Gita; The University of Queensland, School of Public Health
Keywords:	PUBLIC HEALTH, Hypertension < CARDIOLOGY, EPIDEMIOLOGY, Asthma < THORACIC MEDICINE

SCHOLARONE™
Manuscripts

I, the Submitting Author has the right to grant and does grant on behalf of all authors of the Work (as defined in the below author licence), an exclusive licence and/or a non-exclusive licence for contributions from authors who are: i) UK Crown employees; ii) where BMJ has agreed a CC-BY licence shall apply, and/or iii) in accordance with the terms applicable for US Federal Government officers or employees acting as part of their official duties; on a worldwide, perpetual, irrevocable, royalty-free basis to BMJ Publishing Group Ltd ("BMJ") its licensees and where the relevant Journal is co-owned by BMJ to the co-owners of the Journal, to publish the Work in this journal and any other BMJ products and to exploit all rights, as set out in our licence.

The Submitting Author accepts and understands that any supply made under these terms is made by BMJ to the Submitting Author unless you are acting as an employee on behalf of your employer or a postgraduate student of an affiliated institution which is paying any applicable article publishing charge ("APC") for Open Access articles. Where the Submitting Author wishes to make the Work available on an Open Access basis (and intends to pay the relevant APC), the terms of reuse of such Open Access shall be governed by a Creative Commons licence – details of these licences and which Creative Commons licence will apply to this Work are set out in our licence referred to above.

Other than as permitted in any relevant BMJ Author's Self Archiving Policies, I confirm this Work has not been accepted for publication elsewhere, is not being considered for publication elsewhere and does not duplicate material already published. I confirm all authors consent to publication of this Work and authorise the granting of this licence.

For peer review only

Title

Cohort profile: A prospective Australian cohort study of women's reproductive characteristics and risk of chronic disease from menarche to premenopause (M-PreM)

Authors

Hsiu-Wen Chan[1], Shyamali C Dharmage[2], Annette J Dobson[1], Hsin-Fang Chung[1], Deborah Loxton[3], Jenny Doust[1], Grant W Montgomery[4], Emmanuel Stamatakis[5], Rachel R Huxley[6,7], Mark Hamer[8], Jason Abbott[9,10], Bu B Yeap[11,12], Jenny A Visser[13], Harold D McIntyre[14,15], Gregore I Mielke[1], Gita D Mishra[1]

Author affiliations

[1]School of Public Health, The University of Queensland, Brisbane, Queensland, Australia

[2]Allergy and Lung Health Unit, Melbourne School of Population and Global Health, The University of Melbourne, Carlton, Victoria, Australia

[3]Centre for Women's Health Research, School of Medicine and Public Health, University of Newcastle, Newcastle, New South Wales, Australia

[4]The Institute for Molecular Bioscience, The University of Queensland, Brisbane, Queensland, Australia

[5]Charles Perkins Centre, The University of Sydney, Sydney, New South Wales, Australia

[6]Faculty of Health, Deakin University, Burwood, Victoria, Australia

[7]The George Institute for Global Health, University of New South Wales, Sydney, New South Wales, Australia

[8]Division of Surgery and Interventional Sciences, Faculty of Medical Sciences, University College London, United Kingdom

[9]School of Women's and Children's Health, University of New South Wales, Sydney, New South Wales, Australia

[10]Gynaecological Research and Clinical Evaluation (GRACE) Unit, Royal Hospital for Women, Sydney, New South Wales, Australia

[11]Medical School, University of Western Australia, Perth, Western Australia, Australia

[12]Department of Endocrinology and Diabetes, Fiona Stanley Hospital, Perth, Western Australia, Australia

For peer review only

For peer review only - http://bmjopen.bmj.com/site/about/guidelines.xhtml

[13]Department of Internal Medicine, Erasmus MC, University Medical Center Rotterdam, Rotterdam, The Netherlands

[14]School of Medicine, University of Queensland, Brisbane, Queensland, Australia

[15]Mater Research, University of Queensland, Brisbane, Queensland, Australia

Corresponding author

Professor Gita Mishra

The University of Queensland,

School of Public Health

288 Herston Road

Herston QLD 4006

Australia

g.mishra@uq.edu.au

Word count: 3359 words

For peer review only

ABSTRACT

Purpose

Previous studies have identified associations between individual reproductive factors and chronic disease risk among postmenopausal women. However, few have investigated the association of different markers of reproductive function, their interactions, and risk factors of chronic disease among women approaching menopause. The Menarche-to-PreMenopause (M-PreM) study aims to examine the relationship between reproductive factors across the reproductive lifespan and risk indicators for chronic disease among women in their early-to-mid 40s.

Participants

Women born in 1973-78 who participated in the Australian Longitudinal Study on Women's Health (ALSWH) were invited to undertake a clinical or self-administered assessment. A total of 1278 women were recruited from June 2019 to June 2021.

Findings to date

The study measures included functional, cognitive, and cardiometabolic tests, anthropometry, spirometry, and respiratory health questionnaires. Physical activity was also measured continuously over 8 days using a thigh-worn accelerometer. Additionally, a blood sample was collected to measure sex hormones and cardiovascular and metabolic markers, whereas blood and saliva samples were used for the analysis of genetic variants of genes associated with reproductive characteristics and chronic disease. The mean age of the clinic and self-assessed participants was 44.6 and 45.3 years, respectively. The menopausal status of participants was similar between the two arms of the study: 38-41% pre-menopausal, 20% peri-menopausal, and 36% took oral contraception or hormone replacement therapy. Approximately 80% of women had at least one child and participants reported experiencing pregnancy complications: preterm birth (8-13% of pregnancies), gestational diabetes (10%), and gestational hypertension (10-15%).

Future plans

The biomedical data collected in the M-PreM study will be linked to existing ALSWH survey data on sociodemographic factors, health behaviour, reproductive function, and early life factors collected over the

For peer review only - http://bmjopen.bmj.com/site/about/guidelines.xhtml

past 20 years and health administrative data. The association between reproductive factors and risk indicators of chronic disease will be analysed.

KEYWORDS

Chronic disease, reproduction, women's health

STRENGTHS AND LIMITATIONS OF THIS STUDY

- This study is the first to investigate how reproductive function across the reproductive lifespan influences risk indicators for chronic disease amongst women approaching menopause.
- This study collected biomedical and device-measured physical activity data and will use existing survey data from the 1973-78 cohort of the Australian Longitudinal Study on Women's Health, collected since 1996 when women were aged 18-23 years, and linked health administrative data.
- The COVID-19 pandemic adversely impacted participation rates for this study and respiratory data collection.
- Due to the complex blood processing requirements, we were unable to establish study sites convenient for participants living in regional and remote areas.
- Participants who were unable to attend a study site for clinical assessment were posted questionnaires, a physical activity monitor, and a saliva collection kit for self-administered assessment.

INTRODUCTION

Rapidly ageing populations in Australia and globally and the rising tide of associated chronic diseases present a pressing challenge for public health policy. Many age-related chronic diseases show marked sex differences in their prevalence and aetiology, with women being at a greater disadvantage overall. For example, although middle-aged women have a lower prevalence of diabetes[1], women with diabetes are at greater risk of coronary heart disease[2], stroke[3], and all-cause mortality[2] than men with diabetes. After adolescence, the prevalence of asthma is not only higher for Australian women than men across adulthood, but increases rapidly leading up to and through the menopausal transition[4]. Lastly, women have a higher prevalence of non-amnestic mild cognitive impairment than men[5] and experience faster cognitive decline[6].

For peer review only - http://bmjopen.bmj.com/site/about/guidelines.xhtml

There is a growing body of evidence on the links between reproductive factors and risk of chronic disease. Large pooled studies have shown that early menarche, early menopause, and a short reproductive lifespan are associated with increased risk of cardiovascular disease in postmenopausal women[7, 8]. Similarly, meta-analyses and systematic reviews demonstrate associations between early menarche, early menopause, and increased odds of type 2 diabetes[9, 10]. Early menarche is also linked to asthma in early-to-mid adulthood[11].

Few studies have investigated the associations between reproductive health with risk factors of chronic disease in younger women. Early menarche and longer menstrual cycle length (≥33 days) are associated with elevated diastolic blood pressure, total cholesterol, LDL cholesterol, and triglyceride levels among women in their 30s and also those approaching menopause, which may be partially mediated by body composition[12, 13]. Sex hormonal disorders in women of reproductive age, such as polycystic ovary syndrome (PCOS) and premature ovarian insufficiency, have also been linked to cardiometabolic risk factors and/or poor lung function and asthma. Women with PCOS not only have a higher body mass index[14] and waist-to-hip ratio than other women, but they also have higher levels of total and free testosterone and dehydroepiandrosterone (DHEA)[15]. In women with PCOS, there is some evidence that free testosterone is negatively associated with HDL cholesterol levels, whereas DHEA is positively associated with triglyceride and LDL concentration[16]. Sex hormones are also associated with lung function. DHEA is negatively associated with forced expiratory volume in 1 second (FEV1) and forced vital capacity (FVC) and with greater risk of airflow limitation later in life[17]. Other reproductive factors, including early menarche and parity, are associated with poorer lung function (lower FEV1, FVC, and FEV1/FVC; FEV1/FVC is a marker of airway obstruction) and asthma[11, 18, 19].

It has been long recognised that women are at higher risk of developing chronic diseases after menopause, especially cardiometabolic and respiratory disease. However, it is unclear whether reproductive history from adolescence and early-to-mid adulthood affects the risk factors for chronic diseases in the years preceding menopause. Therefore, in order to implement targeted early preventative health strategies, there is a pressing need to examine the relationship between reproductive factors, sex hormones, and risk factors of chronic disease in women before they enter the menopausal transition. Additionally, whilst previous studies

For peer review only - http://bmjopen.bmj.com/site/about/guidelines.xhtml

have identified sex-specific risk factors for chronic conditions, many have viewed reproductive characteristics in isolation, rather than as a series of related and interacting factors.

The Menarche-to-PreMenopause (M-PreM) study aims to:

1) investigate links between reproductive factors and markers of cardiometabolic health, respiratory conditions, and poor cognitive and functional health among women before they reach middle-age, and
2) determine whether body size and physical activity modify these relationships.

The markers of cardiometabolic health measured are glycated haemoglobin (HbA1c), serum lipids, and blood pressure and the markers of respiratory conditions are lung function tests and respiratory health questionnaires. Other measures include functional and cognitive tests. In addition, this study will use existing data on physical and mental health, reproductive factors and sociodemographic data from the Australian Longitudinal Study on Women's Health surveys and linked administrative health records.

COHORT DESCRIPTION

The Australian Longitudinal Study on Women's Health (ALSWH) is a national, prospective study of women's biological and mental health, social and lifestyle factors, and health service use. In 1996, women born in 1921-26, 1945-51, and 1973-78 were randomly selected from the database of Medicare (Australia's universal health insurance scheme) with intentional over-sampling of women from rural and remote areas. Data collection primarily comprises self-report surveys conducted approximately every three years. More information about the ALSWH can be found elsewhere[20, 21].

Exclusion and inclusion criteria

The 8th survey of the 1973-1978 ALSWH cohort was conducted in 2018 when the participants were aged 40-45 years and completed by 7121 participants. In this survey, women were introduced to the M-PreM study and asked to indicate their interest in participating. Those who responded positively (n=4584) were invited to the M-PreM study (Figure 1). The exclusion criteria included women who were pregnant or had been diagnosed with a reproductive cancer and were undergoing treatment at the time of invitation. Chemotherapy

For peer review only - http://bmjopen.bmj.com/site/about/guidelines.xhtml

and radiation treatments for reproductive cancers often affect reproductive function and blood sex hormone concentration[22].

Sample size

The M-PreM study commenced in June 2019 and concluded in June 2021. Due to the SARS-CoV-2 (COVID-19) outbreak in Australia, the study was suspended from March 2020 to November 2020. The operations of this study and participation have been adversely affected by sudden periods of lockdown in different Australian states, border closures, and new health and safety regulations for clinical research. Overall, 1278 participants were recruited: 499 participants had completed a clinical assessment and 779 participants completed a self-administered assessment (Figure 1).

Based on conservative estimates, sample sizes of 499 and 779 for the clinical and self-administered assessments, respectively, provide enough power to detect associations between exposure-outcome (e.g. the association between early menarche and blood pressure) of small to medium magnitudes after adjustment for up to 10 variables using linear regression models. Assuming a power of 80%, a significance level of 5% and a model including covariates that explains 10% of the variation in the outcome (reduced model; R^2 of 0.10), the minimum detectable values for the R^2 difference between the reduced and full models (model including 10 covariates and the exposure of interest) are 0.014 (R^2 of the full model 0.114) and 0.009 (R^2 of the full model: 0.109) for the clinical and self-administered assessments, respectively. For a power of 90%, the minimum detectable values for the R^2 difference between the reduced and full models are 0.019 for the clinical assessment and 0.012 for the self-administered assessment.

DATA COLLECTION AND MEASURES

The M-PreM study will analyse existing longitudinal and linked data from the ALSWH in combination with new biomedical data.

Existing data from the ALSWH

The 1973-78 cohort have completed up to 9 surveys approximately every 3 years between 1996 and 2022. From these surveys, data on socio-demographic factors, self-reported anthropometry, health-related

For peer review only - http://bmjopen.bmj.com/site/about/guidelines.xhtml

For peer review only

behaviours, and reproductive function are available. The specific items relevant for the M-PreM study are detailed in Table 1.

Table 1 Self-reported data from the ALSWH relevant for the M-PreM study

Category	Item*
Sociodemographic factors	Highest educational attainment<br>Area of residence<br>Marital status
Anthropometry (self-reported)	Weight<br>Height<br>Waist circumference
Health-related behaviour	Current and lifetime smoking history<br>Alcohol intake (quantity and frequency)<br>Use of illicit drugs<br>Level of physical activity<br>Sedentary behaviour, self-reported sitting time<br>Dietary intake, food frequency questionnaires
Reproductive function	Age at menarche<br>Dysmenorrhoea<br>Irregular periods<br>Premenstrual syndrome<br>Age at first delivery<br>Number of miscarriages<br>Terminations<br>Stillbirths<br>Live births<br>Diagnosis of endometriosis or polycystic ovary syndrome<br>Use of fertility treatment<br>Use of contraception<br>Hysterectomy and oophorectomy<br>Diagnosed with gestational diabetes or gestational hypertension<br>Premature birth<br>Infant birthweight<br>Duration of breastfeeding
Early life factors	Early life nutrition (breastfeeding) (Adapted from the Longitudinal Study of Australian Children)<br>Mother's pregnancy complications<br>Attitude and behaviour of parents/caregivers during childhood [23]<br>Childhood household condition [24]<br>Childhood health [25]

*Information for the standard questions administered in the ALSWH (not referenced in this table) can be found here[20, 21].

Modes of participation

Study sites were established in the Translational Research Institute (Brisbane), Murdoch Children's Research Institute (Melbourne), Royal Hospital for Women (Sydney), PARC Clinical Research (Adelaide), and Harry Perkins Institute of Medical Research (Perth). Participants were invited to attend a clinical assessment where they undertook a range of biomedical measurements, provided a non-fasting blood sample, were fitted with a

For peer review only

physical activity monitor which they wore for 8 days, and completed an 8-day sleep diary (Table 2). Participants who reported 'ever having PCOS' were also asked to complete a 3-month menstrual diary. Before the COVID-19 pandemic, participants conducted pre- and post-bronchodilator spirometry, however this was replaced by a respiratory health questionnaire after the outbreak emerged for health and safety reasons (more details can be found in the spirometry methods section). Where participants were unable to attend a study site, they were sent a package that allowed collection of self-administered data. This contained an Oragene·DNA saliva collection kit (OG-600; DNA Genotek Inc.), and an accelerometer (Table 2). The package was then returned via Reply Paid post to the study team at the University of Newcastle.

Table 2 Questionnaires, measurements, biological samples collected and biological markers measured.

Category	Clinic	Self-administered
Reproductive characteristics		
Contraception use [20]	✓	✓
Hormone therapy use [20]	✓	✓
Menopausal status [20]	✓	✓
Ever had PCOS	✓	✓
Menstrual cycle staging [26]	✓	✓
3-month menstrual diary (if ever had PCOS)	✓	✓
Respiratory health* (before 13/03/2020)		
Attacks of asthma or wheezy breathing [27, 28]	✓	✓
Use of medications that affect the airways	✓	
Recent respiratory illness	✓	
Spirometry	✓	
Respiratory health* (after 13/03/2020)		
Attacks of asthma or wheezy breathing [27, 28]	✓	✓
Attacks of cough [27, 28]	✓	✓
Phlegm in the chest [27, 28]	✓	✓
History of pneumonia and other chest illnesses [27, 28]	✓	✓
Ever had bronchitis, COPD, and emphysema	✓	✓
COVID-19	✓	✓
Cognition		
Montreal Cognitive Assessment [29]	✓	
Functional tests		
Handgrip strength test [30]	✓	
Chair rise test [31]	✓	
Standing balance test [31]	✓	
Anthropometry		
Height	✓	✓
Weight	✓	✓
Body mass index	✓	✓
Waist circumference	✓	✓
Hip circumference	✓	✓
Arm circumference	✓	✓
Thigh circumference	✓	✓
Physical activity		
ActivPAL accelerometers worn for 8 days [46]	✓	✓

For peer review only - http://bmjopen.bmj.com/site/about/guidelines.xhtml

Sleep patterns		
8-day sleep diary[46]	✓	✓
Biological samples		
Whole blood	✓	
Serum	✓	
Plasma	✓	
Buffy coat	✓	
Saliva		✓
Cardiometabolic tests		
Blood pressure	✓	
Resting heart rate	✓	
Total cholesterol	✓	
HDL cholesterol	✓	
LDL cholesterol	✓	
Glycated haemoglobin (HbA1c)	✓	
Sex hormones		
Estradiol (E2)	✓	
Estrone (E1)	✓	
Testosterone	✓	
Dihydrotestosterone (DHT)	✓	
Progesterone (P)	✓	
Dehydroepiandrosterone (DHEA)	✓	
Anti-Mullerian Hormone (AMH)	✓	
Sex hormone-binding globulin (SHBG)	✓	
Follicle-stimulating hormone (FSH)	✓	
Luteinising hormone (LH)	✓	

PCOS, polycystic ovary syndrome; COPD, chronic obstructive pulmonary disorder.
*The respiratory health questionnaire and measurements changed after the onset of the COVID-19 pandemic.

Questionnaires

Reproductive characteristics, including use of contraception and hormone therapy, menopausal status, and self-reported polycystic ovary syndrome (PCOS), have been captured using questions from the ALSWH surveys[20]. Menstrual cycle stage at the time of assessment was determined using questions from the Seattle Midlife Women's Health Study[26]. Respiratory health, including attacks of asthma or wheezy breathing, whistling in the chest, shortness of breath and chest tightness was determined using the questionnaire from the Follow-up of the Tasmanian Longitudinal Health Study from first to sixth decade[27], which was adapted from the European Community Respiratory Health Survey II [28]. Cognition was assessed using the Montreal Cognitive Assessment[29].

Handgrip strength test

Handgrip strength was measured (in kilograms) using Jamar hydraulic hand dynamometer (model 5030J1)[30]. Participants were seated with a straight-back posture and their feet flat on the ground. They

For peer review only

For peer review only - http://bmjopen.bmj.com/site/about/guidelines.xhtml

were asked to hold their elbow against their side at a 90° angle and their forearm and wrist in a neutral position. The handgrip strength test was performed once in each hand.

Chair rise test

After removing their shoes, participants were asked to sit in a straight-backed armless chair with a horizontal, flat seat. Participants were assessed on the length of time (in seconds) taken to complete 10 sets of a sit-stand-sit manoeuvre[31].

Standing balance test

Participants were tested for the length of time (in seconds) that they could maintain a one-legged stance for a maximum of 30 seconds, firstly with their eyes open and then closed[31]. This test was performed without shoes and with both arms crossed over the chest. Participants were asked to stand on their preferred foot and to raise their non-preferred foot off the floor by bending their knee.

Blood pressure and heart rate at rest

After resting in a seated position for 5 minutes, three measurements of systolic and diastolic blood pressures and heart rate, interspersed by 1-2 minutes breaks, was recorded using an automated blood pressure monitor. For analysis, only the second and third measurements were averaged, as recommended by the World Health Organization protocol[32].

Anthropometric assessment

Weight without shoes was measured once using a digital scale to the nearest 0.1kg. Height without shoes was measured once using a stadiometer to the nearest millimetre. Using these measurements, body mass index (kg/m^2) was calculated. The waist circumference was measured at the midpoint between the bottom of the last palpable rib and top of the iliac crest (for self-assessment, this was described as ‘the midpoint between bottom of the ribs and top of the hip’). The hip circumference was measured at the widest point of the buttocks (for self-assessment, this was described as the ‘widest portion of the bottom’). The arm circumference was measured at the midpoint between the acromion process of the scapula and the olecranon process (for self-assessment, this was described as the ‘midpoint between the top of your shoulder to the point of your elbow’). Thigh circumference was measured at the midpoint between the lateral superior margin of the patella and the anterior superior iliac spine (for self-assessment, this was described as the

For peer review only - http://bmjopen.bmj.com/site/about/guidelines.xhtml

'midpoint between the top of your hip to the top of your knee'). Circumference measurements in the clinic were conducted to the nearest millimetre in duplicate, whereas at home, participants were asked to record the measurements in centimetres to one decimal point. Where the difference between the two measurements exceeded 5mm, a third measurement was performed. The average of the two or three measurements was then calculated.

Spirometry

Lung function testing was conducted from 9 July 2019 to 13 March 2020, after which it was removed from the protocol due to health and safety concerns as a result of the COVID-19 pandemic. Lung function testing using Easy-on-PC spirometers (NDD Medizintechnik AG, Switzerland) was performed according to the American Thoracic Society and European Respiratory Society guidelines[33] and based on the protocols of the Tasmanian Longitudinal Health Study[27]. Participants were required to perform a minimum of three acceptable trials for each pre- and post-bronchodilator test. Post-bronchodilator spirometry was performed 10-15 minutes after the bronchodilator, salbutamol (300μg), was administered via a spacer.

To replace the lung function testing in the context of the COVID-19 pandemic, an extended respiratory health questionnaire was then given to all participants who were recruited after the M-PreM study resumed on 10 September 2020. In addition to the original respiratory health questions (detailed in the Questionnaires section), the following questions were asked: ever had and age of diagnosis of bronchitis, chronic obstructive pulmonary disorder, and emphysema; and history of bronchitis, phlegm in the chest, cough in the absence of a cold, cough with phlegm, and chest illnesses including pneumonia. Like the original respiratory health questions, these questions were also sourced from the Follow-up of the Tasmanian Longitudinal Health Study from first to sixth decade[27]. Lastly, questions about COVID-19 testing and results as well as self-reported changes to participant's respiratory function were in the extended respiratory health questionnaire.

Participants who had already completed a clinical or self-administered assessment prior to the onset of the COVID-19 pandemic were also invited to complete the extended respiratory health questionnaire.

For peer review only - http://bmjopen.bmj.com/site/about/guidelines.xhtml

Physical activity and sleep patterns

Accelerometers (activPAL3 micro and activPAL4 micro devices; PAL Technologies, UK) were attached to the middle of the anterior surface of the right thigh using adhesive dressing (3M Tegaderm) and worn continuously for 8 full days. Participants were asked to log their sleep patterns (bedtime, estimated time they fell asleep, waking time, and time they got out of bed) and sleep quality (number of night wakings, how well they slept) for 8 days.

Biological samples and biomarker testing

BD Vacutainer® EDTA (for plasma and HbA1c testing), SST II (for serum and cholesterol testing), and plain tubes (for serum) were used to collect non-fasting venous blood. Whole blood, serum, plasma and buffy coat were also extracted. All samples are biobanked at -80°C at the Institute of Molecular Biosciences (Brisbane, Australia).

Serum estrone (E1), estradiol (E2), testosterone, dihydrotestosterone (DHT), progesterone (P), and dehydroepiandrosterone (DHEA) concentrations were analysed using a validated liquid chromatography-mass spectrometry (LC-MS) method by the Andrology Laboratory at the ANZAC Research Institute (Sydney, Australia)[34, 35]. Serum sex hormone-binding globulin (SHBG), follicle-stimulated hormone (FSH), and luteinizing hormone (LH) concentrations were analysed by the Concord Repatriation General Hospital Diagnostic Pathology Unit (Sydney, Australia) using commercial immunoassays (Roche). Anti-Mullerian Hormone concentration in serum will be measured by NSW Health Pathology (Sydney, Australia). Total, LDL-, and HDL-cholesterol and glycated haemoglobin A1c (HbA1c) was measured by Pathology Queensland (Central Laboratory, Brisbane, Australia) using routine auto-analyser methods. In the future, DNA will be extracted from the stored blood and saliva samples to identify genetic factors associated with reproductive factors, and cardiometabolic and respiratory conditions. The differences in samples should not affect DNA sequences, but could possibly affect epigenetic markers.

Patient and public involvement

A pilot study was conducted to develop and test the study protocol and provided an opportunity for participant feedback.

For peer review only - http://bmjopen.bmj.com/site/about/guidelines.xhtml

FINDINGS TO DATE

A total of 1278 participants were recruited to the M-PreM study. Of these, 499 attended a clinical study site and 779 completed a self-assessment. The demographic characteristics of these participants were based on their responses in the 8th survey of the ALSWH (Table 3) and compared with the remaining cohort (non-participants). As expected, those who completed a clinical assessment predominantly lived in a major city whereas those who participated in the self-assessment were spread amongst major cities, and inner and outer regional areas. Compared with the participants who completed the self-assessment or did not participate in the M-PreM study, in-clinic participants were more likely to be university-educated and less likely to have 3 or more children, to ever have smoked or have a history of preterm birth. They also started having children at a later age. Participants in the M-PreM study, regardless of mode of participation, were more likely to report better health and be non-drinkers than non-participants.

Table 3 Characteristics of the M-PreM participants and the remainder of the ALSWH 1973-78 cohort.

	M-PreM Clinical assessment n=499 (7.01%)	M-PreM Self-assessment n=779 (10.94%)	1973-78 ALSWH Survey 8 cohort, not in M-PreM n=5843 (82.05%)	p-value*
Area of residence, n (%)				
Major city	463 (93.16)	313 (40.44)	3248 (57.40)	<0.0001
Inner regional	26 (5.23)	275 (35.53)	1594 (28.17)	
Outer regional	4 (0.80)	164 (21.19)	695 (12.28)	
Remote	4 (0.80)	22 (2.84)	122 (2.16)	
Missing	2	5	184	
Highest qualification, n (%)				
Less than year 12 or equiv.	10 (2.00)	39 (5.01)	305 (5.55)	<0.0001
Year 12 or equiv.	24 (4.81)	68 (8.73)	529 (9.62)	
Trade/apprenticeship/Cert/Dip	92 (18.44)	229 (29.40)	1586 (28.85)	
University	373 (74.75)	443 (56.87)	3078 (55.98)	
Missing	0	0	345	
Marital status, n (%)				
Never married	58 (11.62)	77 (9.88)	636 (11.55)	0.771
Married/De facto	390 (78.16)	624 (80.10)	4299 (78.08)	
Separated	26 (5.21)	33 (4.24)	236 (4.29)	
Divorced	22 (4.41)	40 (5.13)	309 (5.61)	
Widowed	3 (0.60)	5 (0.64)	26 (0.47)	
Missing	0	0	337	
Employment status, n (%)				
Not in labour force	60 (12.02)	103 (13.24)	840 (14.89)	0.028
Employed full time	225 (45.09)	330 (42.42)	2529 (44.82)	
Employed part time	180 (36.07)	274 (35.22)	1752 (31.05)	
Employed casually	34 (6.81)	71 (9.13)	521 (9.23)	
Missing	0	1	201	
Self-reported health, n (%)				
Excellent/very good	300 (60.12)	477 (61.23)	3130 (53.89)	0.0002
Good	157 (31.46)	220 (28.24)	2003 (34.49)	
Fair/poor	42 (8.42)	82 (10.53)	675 (11.62)	

For peer review only - http://bmjopen.bmj.com/site/about/guidelines.xhtml

Missing	0	0	35	
Smoking status, n (%)				
Never smoked	344 (68.94)	482 (61.95)	3428 (61.00)	0.0002
Ex-smoker	125 (25.05)	235 (30.21)	1587 (28.24)	
Current smoker	30 (6.01)	61 (7.84)	605 (10.77)	
Missing	0	1	223	
Alcohol use, n (%)				
Non-drinker	39 (7.82)	64 (8.23)	609 (10.82)	0.014
Low risk	432 (86.57)	663 (85.22)	4598 (81.73)	
High risk	28 (5.61)	51 (6.56)	419 (7.45)	
Missing	0	1	217	
Number of children, n (%)				
No children	105 (21.04)	127 (16.30)	1149 (19.66)	0.005
1 child	56 (11.22)	96 (12.32)	789 (13.50)	
2 children	229 (45.89)	345 (44.29)	2328 (39.84)	
3 or more children	109 (21.84)	211 (27.09)	1577 (26.99)	
Missing	0	0	0	
Age at menarche, n (%)				
11 years or younger	52 (12.53)	74 (11.23)	551 (11.51)	0.749
12 years	115 (27.71)	185 (28.07)	1371 (28.63)	
13 years	124 (29.88)	202 (30.65)	1482 (30.95)	
14 years	80 (19.28)	106 (16.08)	813 (16.98)	
15 years or older	44 (10.60)	92 (13.96)	571 (11.93)	
Missing	84	120	1055	
History of preterm birth, n (%)				
Yes	33 (8.38)	87 (13.43)	668 (14.49)	0.003
No	361 (91.62)	561 (86.57)	3943 (85.51)	
Missing	105	131	1232	
History of stillbirth, n (%)				
Yes	11 (2.79)	7 (1.07)	92 (1.96)	0.128
No	383 (97.21)	645 (98.93)	4593 (98.04)	
Missing	105	127	1158	
History of gestational diabetes (%)				
Yes	39 (9.90)	64 (9.89)	490 (10.63)	0.782
No	355 (91.10)	583 (90.11)	4119 (89.37)	
Missing	105	132	1234	
History of gestational hypertension (%)				
Yes	39 (9.92)	98 (15.15)	646 (14.02)	0.0476
No	354 (90.08)	549 (84.85)	3963 (85.98)	
Missing	106	132	1234	
Age at first birth, mean (SD)	31.31 (4.92)	29.67 (5.16)	29.59 (5.28)	<0.0001

*Analysis was conducted using ANOVA and Chi-square tests.

Table 4 shows some of the outcome measurements. At the time of participation, approximately 40% of the women were premenopausal and 20% had begun their menopausal transition, regardless of their mode of participation. Over 55% of participants were categorised as overweight or obese based on their body mass index (BMI). The BMI and body measurements were similar between the clinic- and self-assessed participants, except arm and thigh circumference, which were larger in clinic participants. Also, the prevalence of respiratory symptoms was similar between the clinic- and self-assessed participants.

For peer review only

For peer review only - http://bmjopen.bmj.com/site/about/guidelines.xhtml

Table 4 Clinical outcomes from the Menarche-to-PreMenopause study.

	Clinic assessment (n=499)		Self-assessment (n=779)		p-value
	n		n		
Age (years), mean (SD)	499	44.60 (1.61)	779	45.28 (1.78)	<0.001
Menopausal status, %					
Surgical menopause	8	1.61	20	2.73	0.531
Not defined due to HRT or OCP use	177	35.61	263	35.93	
Pre-menopausal	202	40.64	275	37.57	
Peri-menopausal	99	19.92	152	20.77	
Post-menopausal	11	2.21	22	3.01	
Missing	2		47		
Body mass index category, %					
Underweight <18.5kg/m^2)	2	0.40	8	1.10	0.380
Normal weight (18.5 to <25kg/m^2)	202	40.56	311	42.60	
Overweight (25 to <30kg/m^2)	147	29.52	193	26.44	
Obese (≥30kg/m^2)	147	29.52	218	29.86	
Missing	1		49		
Body mass index, mean (SD)	498	27.80 (6.43)	730	27.68 (6.48)	0.750
Body measurements (cm), mean (SD)					
Waist circumference	499	88.78 (14.84)	748	89.18 (14.98)	0.647
Hip circumference	498	106.21 (13.49)	749	105.97 (13.62)	0.755
Arm circumference	498	31.27 (5.13)	736	30.36 (4.35)	0.001
Thigh circumference	498	59.06 (8.47)	746	55.74 (7.78)	<0.001
Blood Pressure (mmHg), mean (SD)					
Systolic	494	119.11 (12.37)	-	-	n/a
Diastolic	494	77.29 (8.84)	-	-	
Resting heart rate (beats per min), mean (SD)	493	75.15 (10.50)	-	-	n/a
Cardiometabolic biomarkers, mean (SD)					
Total cholesterol (mmol/L)	489	5.21 (0.89)	-	-	n/a
HDL cholesterol (mmol/L)	489	1.64 (0.39)	-	-	
LDL cholesterol (mmol/L)	486	3.00 (0.77)	-	-	
HbA1c (%)	489	5.14 (0.36)	-	-	
Handgrip strength (kg), mean (SD)					n/a
Dominant hand	486	30.57 (6.01)	-	-	
Non-dominant hand	486	28.62 (5.26)	-	-	
Standing balance test (seconds)*, mean (SD)					n/a
Eyes open	498	28.57 (5.26)	-	-	
Eyes closed	498	10.97 (10.36)	-	-	
Chair rise test (seconds), mean (SD)	495	16.72 (4.77)	-	-	n/a
Reciprocal of the chair rise test**, mean (SD)	495	6.48 (1.86)			
Montreal Cognitive Assessment***, mean (SD)	499	27.69 (1.94)	-	-	n/a
Lung function, prebronchodilator, mean (SD)					
FEV1 (L)	283	3.03 (0.45)	-	-	n/a
FVC (L)	283	3.84 (0.56)	-	-	
FEV1/FVC (%)	283	0.79 (0.05)	-	-	
Lung function, postbronchodilator, mean (SD)					
FEV1 (L)	283	3.12 (0.46)	-	-	n/a
FVC (L)	283	3.84 (0.56)	-	-	
FEV1/FVC (%)	283	0.82 (0.05)	-	-	
Ever had asthma or wheezy breathing (%)					
Yes	194	38.96	279	36.23	0.328
No	304	61.04	491	63.77	
Missing	1		9		
Had asthma or wheezy breathing in the last 12 months (%)					
Yes	82	42.27	139	49.12	0.141
No	112	57.73	144	50.88	
Missing	305		496		

For peer review only

For peer review only - http://bmjopen.bmj.com/site/about/guidelines.xhtml

Wheeze or whistling in your chest in the last 12 months (%)					
Yes	79	15.86	137	17.77	0.378
No	419	84.14	634	82.23	
Missing	1		8		
Ever had attacks of cough with phlegm (%)					
Yes	239	51.84	361	51.87	0.994
No	222	48.16	335	48.13	
Missing	38		83		
Ever had cough in the absence of a cold (%)					
Yes	116	25.16	141	20.17	0.045
No	345	74.84	558	79.83	
Missing	38		80		
Ever had phlegm in your chest in the absence of a cold (%)					
Yes	28	6.07	49	7.01	0.531
No	433	93.93	650	92.99	
Missing	38		80		

Analysis was conducted using t-tests and Chi-square tests.
*Maximum score of 30 seconds
** Reciprocal of chair rise time x 100
**Score out of 30

STRENGTHS AND LIMITATIONS

The strength of the M-PreM study is that it presents an invaluable opportunity to investigate the relationship between reproductive characteristics across the reproductive lifespan and risk indicators for chronic disease and functional and mental health before midlife. In spite of the difficult circumstances due to the COVID-19 pandemic, we have succeeded in recruiting a large sample of participants who have completed questionnaires, clinical measures, physical activity assessments, and blood sample collection to augment the existing information available from the ALSWH surveys. The distributions of measurements were well within the expected ranges found in other studies for handgrip strength[36], body mass index[37], systolic and diastolic blood pressure[37, 38], cholesterol[39, 40], waist circumference[37], hip circumference[41, 42], thigh circumference[41, 42], and arm circumference[43]. The mean Montreal Cognitive Assessment scores was higher in our cohort compared with other studies[44, 45], possibly due to the high representation of women with university education in our cohort.

The conduct of the study was affected by the coronavirus (COVID-19) pandemic. After two lengthy study suspensions, the participation rate was lower than expected as participants were uncertain about attending hospitals where the study sites were established and people continued to work from home and juggle child care responsibilities, even after the government lifted stay-at-home orders. COVID-19 outbreaks

For peer review only - http://bmjopen.bmj.com/site/about/guidelines.xhtml

occurred sporadically throughout the study period and study sites were either closed at short notice or their establishment was halted. In addition, the study protocol was modified to minimise risk of COVID-19 transmission between study personnel and participants. Specifically, the objective lung function testing was replaced by a self-reported respiratory health questionnaire. Participation was also affected by the 2019-2020 Australian bushfire disaster, which prevented a study site from being established in Canberra. Another limitation was the location of the study sites. Although ALSWH is a national study, for M-PreM, study sites could only be established in major cities.

Regardless of the limitations, the study will have wealth of data on potentially sufficient number of participants to investigate all the major research questions, which will make a substantial contribution to understanding female reproductive health.

FURTHER DETAILS

Ethics approval and consent to participate

The study has been approved by the Metro South Health and Health Services Human Research Ethics Committee (Brisbane, Australia; Ref: HREC/2019/QMS/52052) and ratified by the University of Newcastle and the University of Queensland Human Research Ethics Committees. All participants provided informed consent by completing an electronic or paper participant consent form.

Data availability statement

Data are available upon reasonable request. Access to the M-PreM dataset requires approval from the Australian Longitudinal Study on Women's Health Data Access Committee. More information can be found at the ALSWH website: https://alswh.org.au/for-data-users/.

Funding

This work is supported by the National Health and Medical Research Council Project Grant (APP1129592). GM is supported by an NHMRC Principal Research Fellowship (APP1121844).

Author contributions

For peer review only - http://bmjopen.bmj.com/site/about/guidelines.xhtml

GM designed the study, is the lead applicant on the funding application. SCD, JAV, DL, RRH, AJD, GWM, HDM, and JD are co-applicants of the funding application and provided feedback on the design and the operations of the study. SCD, JA, and BBY were study site principal investigators and provided on-site supervision. ES provided accelerometers and protocols for physical activity data collection. H-WC is the project coordinator for the study and drafted this manuscript. H-FC is the postdoctoral fellow for this study and provided feedback on the study operations. GIM is a postdoctoral fellow for this study and provided statistical expertise. All authors reviewed and approved the final manuscript.

Acknowledgements

We would like to acknowledge the Australian Government Department of Health for funding the Australian Longitudinal Study on Women's Health and the women who provided survey data and biomedical data for this study.

Consent for publication

Not applicable

Competing interests

The authors declare no competing interests.

REFERENCES

1. Saeedi P, Petersohn I, Salpea P, et al. Global and regional diabetes prevalence estimates for 2019 and projections for 2030 and 2045: Results from the International Diabetes Federation Diabetes Atlas, 9(th) edition. *Diabetes Res Clin Pract* 2019;157:107843.

2. Wang Y, O'Neil A, Jiao Y, et al. Sex differences in the association between diabetes and risk of cardiovascular disease, cancer, and all-cause and cause-specific mortality: a systematic review and meta-analysis of 5,162,654 participants. *BMC Med* 2019;17(1):136.

3. Peters SA, Huxley RR, Woodward M. Diabetes as a risk factor for stroke in women compared with men: a systematic review and meta-analysis of 64 cohorts, including 775,385 individuals and 12,539 strokes. *Lancet* 2014;383(9933):1973-80.

4. Leynaert B, Sunyer J, Garcia-Esteban R, et al. Gender differences in prevalence, diagnosis and incidence of allergic and non-allergic asthma: a population-based cohort. *Thorax* 2012;67(7):625-31.

For peer review only - http://bmjopen.bmj.com/site/about/guidelines.xhtml

For peer review only

5. Au B, Dale-McGrath S, Tierney MC. Sex differences in the prevalence and incidence of mild cognitive impairment: A meta-analysis. *Ageing Res Rev* 2017;35:176-99.

6. Levine DA, Gross AL, Briceno EM, et al. Sex Differences in Cognitive Decline Among US Adults. *JAMA Netw Open* 2021;4(2):e210169.

7. Zhu D, Chung HF, Dobson AJ, et al. Age at natural menopause and risk of incident cardiovascular disease: a pooled analysis of individual patient data. *Lancet Public Health* 2019;4(11):e553-e64.

8. Mishra SR, Chung HF, Waller M, et al. Association Between Reproductive Life Span and Incident Nonfatal Cardiovascular Disease: A Pooled Analysis of Individual Patient Data From 12 Studies. *JAMA Cardiol* 2020;5(12):1410-8.

9. Cheng TS, Day FR, Lakshman R, et al. Association of puberty timing with type 2 diabetes: A systematic review and meta-analysis. *PLoS Med* 2020;17(1):e1003017.

10. Anagnostis P, Christou K, Artzouchaltzi AM, et al. Early menopause and premature ovarian insufficiency are associated with increased risk of type 2 diabetes: a systematic review and meta-analysis. *Eur J Endocrinol* 2019;180(1):41-50.

11. Lieberoth S, Gade EJ, Brok J, et al. Age at menarche and risk of asthma: systematic review and meta-analysis. *J Asthma* 2014;51(6):559-65.

12. Bubach S, Horta BL, Goncalves H, et al. Early age at menarche and metabolic cardiovascular risk factors: mediation by body composition in adulthood. *Sci Rep* 2021;11(1):148.

13. Matthews KA, Santoro N, Lasley B, et al. Relation of cardiovascular risk factors in women approaching menopause to menstrual cycle characteristics and reproductive hormones in the follicular and luteal phases. *J Clin Endocrinol Metab* 2006;91(5):1789-95.

14. Teede HJ, Joham AE, Paul E, et al. Longitudinal weight gain in women identified with polycystic ovary syndrome: results of an observational study in young women. *Obesity (Silver Spring)* 2013;21(8):1526-32.

15. Perez-Lopez FR, Ornat L, Lopez-Baena MT, et al. Circulating kisspeptin and anti-mullerian hormone levels, and insulin resistance in women with polycystic ovary syndrome: A systematic review, meta-analysis, and meta-regression. *Eur J Obstet Gynecol Reprod Biol* 2021;260:85-98.

16. Amiri M, Tehrani FR, Bidhendi-Yarandi R, et al. Relationships Between Biochemical Markers of Hyperandrogenism and Metabolic Parameters in Women with Polycystic Ovary Syndrome: A Systematic Review and Meta-Analysis. *Horm Metab Res* 2019;51(1):22-34.

17. Pesce G, Triebner K, van der Plaat DA, et al. Low serum DHEA-S is associated with impaired lung function in women. *EClinicalMedicine* 2020;23:100389.

For peer review only - http://bmjopen.bmj.com/site/about/guidelines.xhtml

18. Tang R, Fraser A, Magnus MC. Female reproductive history in relation to chronic obstructive pulmonary disease and lung function in UK biobank: a prospective population-based cohort study. *BMJ Open* 2019;9(10):e030318.

19. Campbell B, Simpson JA, Bui DS, et al. Early menarche is associated with lower adult lung function: A longitudinal cohort study from the first to sixth decade of life. *Respirology* 2020;25(3):289-97.

20. Dobson AJ, Hockey R, Brown WJ, et al. Cohort Profile Update: Australian Longitudinal Study on Women's Health. *Int J Epidemiol* 2015;44(5):1547,a-f.

21. Lee C, Dobson AJ, Brown WJ, et al. Cohort Profile: the Australian Longitudinal Study on Women's Health. *Int J Epidemiol* 2005;34(5):987-91.

22. Meirow D, Nugent D. The effects of radiotherapy and chemotherapy on female reproduction. *Hum Reprod Update* 2001;7(6):535-43.

23. Steptoe A, Breeze E, Banks J, et al. Cohort profile: the English longitudinal study of ageing. *Int J Epidemiol* 2013;42(6):1640-8.

24. Kraal AZ, Dotterer HL, Sharifian N, et al. Physical Activity in Early- and Mid-Adulthood Are Independently Associated With Longitudinal Memory Trajectories in Later Life. *J Gerontol A Biol Sci Med Sci* 2021;76(8):1495-503.

25. Pakpahan E, Hoffmann R, Kroger H. Retrospective life course data from European countries on how early life experiences determine health in old age and possible mid-life mediators. *Data Brief* 2017;10:277-82.

26. Mitchell ES, Woods NF, Mariella A. Three stages of the menopausal transition from the Seattle Midlife Women's Health Study: toward a more precise definition. *Menopause* 2000;7(5):334-49.

27. Tan DJ, Walters EH, Perret JL, et al. Clinical and functional differences between early-onset and late-onset adult asthma: a population-based Tasmanian Longitudinal Health Study. *Thorax* 2016;71(11):981-7.

28. European Community Respiratory Health Survey IISC. The European Community Respiratory Health Survey II. *Eur Respir J* 2002;20(5):1071-9.

29. Nasreddine ZS, Phillips NA, Bedirian V, et al. The Montreal Cognitive Assessment, MoCA: a brief screening tool for mild cognitive impairment. *J Am Geriatr Soc* 2005;53(4):695-9.

30. Spruit MA, Sillen MJ, Groenen MT, et al. New normative values for handgrip strength: results from the UK Biobank. *J Am Med Dir Assoc* 2013;14(10):775 e5-11.

For peer review only - http://bmjopen.bmj.com/site/about/guidelines.xhtml

31. Kuh D, Bassey EJ, Butterworth S, et al. Grip strength, postural control, and functional leg power in a representative cohort of British men and women: associations with physical activity, health status, and socioeconomic conditions. *J Gerontol A Biol Sci Med Sci* 2005;60(2):224-31.

32. World Health Organization. WHO technical specifications for automated non-invasive blood pressure measuring devices with cuff. World Health Organization. 2020.

33. Miller MR, Hankinson J, Brusasco V, et al. Standardisation of spirometry. *Eur Respir J* 2005;26(2):319-38.

34. Harwood DT, Handelsman DJ. Development and validation of a sensitive liquid chromatography-tandem mass spectrometry assay to simultaneously measure androgens and estrogens in serum without derivatization. *Clin Chim Acta* 2009;409(1-2):78-84.

35. Handelsman DJ, Gibson E, Davis S, et al. Ultrasensitive Serum Estradiol Measurement by Liquid Chromatography-Mass Spectrometry in Postmenopausal Women and Mice. *J Endocr Soc* 2020;4(9):bvaa086.

36. Bohannon RW, Wang YC, Yen SC, et al. Handgrip Strength: A Comparison of Values Obtained From the NHANES and NIH Toolbox Studies. *Am J Occup Ther* 2019;73(2):7302205080p1-p9.

37. Statistics ABo. Data from: National Health Survey: First Results, 2017-18, December 12, 2018.

38. Mitchell GF, Wang N, Palmisano JN, et al. Hemodynamic correlates of blood pressure across the adult age spectrum: noninvasive evaluation in the Framingham Heart Study. *Circulation* 2010;122(14):1379-86.

39. Shelley JM, Green A, Smith AM, et al. Relationship of endogenous sex hormones to lipids and blood pressure in mid-aged women. *Ann Epidemiol* 1998;8(1):39-45.

40. Szoeke C, Lehert P, Henderson VW, et al. Predictive Factors for Verbal Memory Performance Over Decades of Aging: Data from the Women's Healthy Ageing Project. *Am J Geriatr Psychiatry* 2016;24(10):857-67.

41. Koskova I, Petrasek R, Vondra K, et al. Weight, body composition and fat distribution changes of Czech women in the different reproductive phases: a longitudinal study. *Prague Med Rep* 2007;108(3):226-42.

42. Kuk JL, Janiszewski PM, Ross R. Body mass index and hip and thigh circumferences are negatively associated with visceral adipose tissue after control for waist circumference. *Am J Clin Nutr* 2007;85(6):1540-4.

43. Soriguer F, Morcillo S, Hernando V, et al. Type 2 diabetes mellitus and other cardiovascular risk factors are no more common during menopause: longitudinal study. *Menopause* 2009;16(4):817-21.

For peer review only

For peer review only - http://bmjopen.bmj.com/site/about/guidelines.xhtml

44. Rossetti HC, Lacritz LH, Cullum CM, et al. Normative data for the Montreal Cognitive Assessment (MoCA) in a population-based sample. *Neurology* 2011;77(13):1272-5.

45. Santangelo G, Siciliano M, Pedone R, et al. Normative data for the Montreal Cognitive Assessment in an Italian population sample. *Neurol Sci* 2015;36(4):585-91.

46. Hamer M, Stamatakis E, Chastin S, et al. Feasibility of Measuring Sedentary Time Using Data From a Thigh-Worn Accelerometer. *Am J Epidemiol* 2020;189(9):963-71.

FIGURE LEGENDS

Figure 1 The participant recruitment flow chart for the M-PreM study.

LICENCE STATEMENT

I, the Submitting Author has the right to grant and does grant on behalf of all authors of the Work (as defined in the below author licence), an exclusive licence and/or a non-exclusive licence for contributions from authors who are: i) UK Crown employees; ii) where BMJ has agreed a CC-BY licence shall apply, and/or iii) in accordance with the terms applicable for US Federal Government officers or employees acting as part of their official duties; on a worldwide, perpetual, irrevocable, royalty-free basis to BMJ Publishing Group Ltd ("BMJ") its licensees and where the relevant Journal is co-owned by BMJ to the co-owners of the Journal, to publish the Work in BMJ Open and any other BMJ products and to exploit all rights, as set out in our licence.

The Submitting Author accepts and understands that any supply made under these terms is made by BMJ to the Submitting Author unless you are acting as an employee on behalf of your employer or a postgraduate student of an affiliated institution which is paying any applicable article publishing charge ("APC") for Open Access articles. Where the Submitting Author wishes to make the Work available on an Open Access basis (and intends to pay the relevant APC), the terms of reuse of such Open Access shall be governed by a Creative Commons licence – details of these licences and which Creative Commons licence will apply to this Work are set out in our licence referred to above.

For peer review only

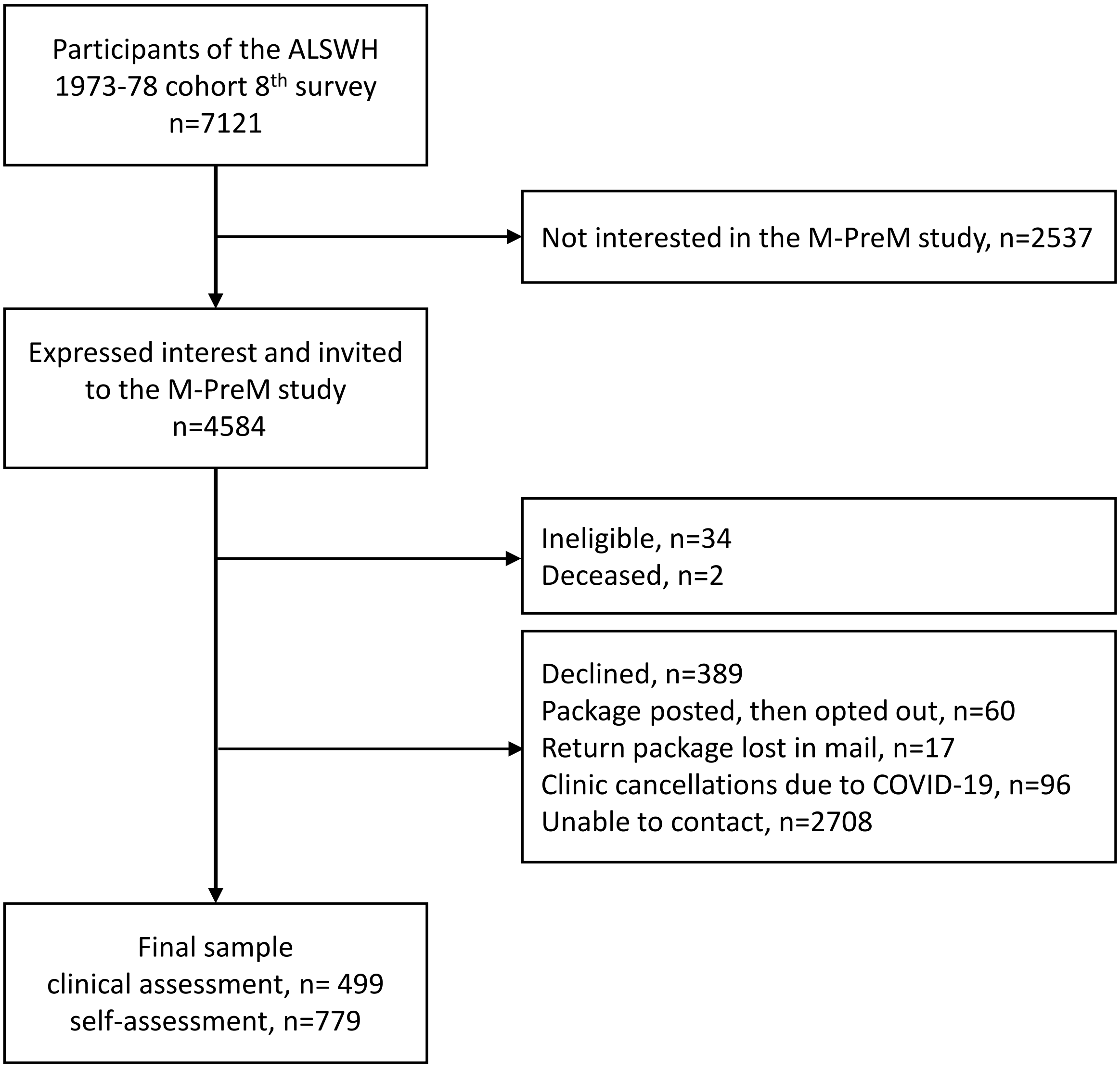
Participants of the ALSWH
1973-78 cohort 8th survey
n=7121
Not interested in the M-PreM study, n=2537
Expressed interest and invited
to the M-PreM study
n=4584
Ineligible, n=34
Deceased, n=2
Declined, n=389
Package posted, then opted out, n=60
Return package lost in mail, n=17
Clinic cancellations due to COVID-19, n=96
Unable to contact, n=2708
Final sample
clinical assessment, n= 499
self-assessment, n=779

STROBE Statement—Checklist of items that should be included in reports of ***cohort studies***

	Item No	Recommendation	Page No
**Title and abstract**	1	(*a*) Indicate the study's design with a commonly used term in the title or the abstract	1
		(*b*) Provide in the abstract an informative and balanced summary of what was done and what was found	3
**Introduction**			
Background/rationale	2	Explain the scientific background and rationale for the investigation being reported	4-5
Objectives	3	State specific objectives, including any prespecified hypotheses	6
**Methods**			
Study design	4	Present key elements of study design early in the paper	6
Setting	5	Describe the setting, locations, and relevant dates, including periods of recruitment, exposure, follow-up, and data collection	7-10
Participants	6	(*a*) Give the eligibility criteria, and the sources and methods of selection of participants. Describe methods of follow-up	6-7
		(*b*) For matched studies, give matching criteria and number of exposed and unexposed	n/a
Variables	7	Clearly define all outcomes, exposures, predictors, potential confounders, and effect modifiers. Give diagnostic criteria, if applicable	7-13
Data sources/ measurement	8*	For each variable of interest, give sources of data and details of methods of assessment (measurement). Describe comparability of assessment methods if there is more than one group	7-13
Bias	9	Describe any efforts to address potential sources of bias	n/a
Study size	10	Explain how the study size was arrived at	6-7
Quantitative variables	11	Explain how quantitative variables were handled in the analyses. If applicable, describe which groupings were chosen and why	n/a
Statistical methods	12	(*a*) Describe all statistical methods, including those used to control for confounding	n/a
		(*b*) Describe any methods used to examine subgroups and interactions	n/a
		(*c*) Explain how missing data were addressed	n/a
		(*d*) If applicable, explain how loss to follow-up was addressed	n/a
		(*e*) Describe any sensitivity analyses	n/a
**Results**			
Participants	13*	(a) Report numbers of individuals at each stage of study—eg numbers potentially eligible, examined for eligibility, confirmed eligible, included in the study, completing follow-up, and analysed	6-7
		(b) Give reasons for non-participation at each stage	6-7
		(c) Consider use of a flow diagram	6-7
Descriptive data	14*	(a) Give characteristics of study participants (eg demographic, clinical, social) and information on exposures and potential confounders	14-15
		(b) Indicate number of participants with missing data for each variable of interest	14-15
		(c) Summarise follow-up time (eg, average and total amount)	n/a
Outcome data	15*	Report numbers of outcome events or summary measures over time	16-17

For peer review only - http://bmjopen.bmj.com/site/about/guidelines.xhtml

Main results	16	(*a*) Give unadjusted estimates and, if applicable, confounder-adjusted estimates and their precision (eg, 95% confidence interval). Make clear which confounders were adjusted for and why they were included	n/a
		(*b*) Report category boundaries when continuous variables were categorized	16-17
		(*c*) If relevant, consider translating estimates of relative risk into absolute risk for a meaningful time period	n/a
Other analyses	17	Report other analyses done—eg analyses of subgroups and interactions, and sensitivity analyses	n/a
**Discussion**			
Key results	18	Summarise key results with reference to study objectives	n/a
Limitations	19	Discuss limitations of the study, taking into account sources of potential bias or imprecision. Discuss both direction and magnitude of any potential bias	17-18
Interpretation	20	Give a cautious overall interpretation of results considering objectives, limitations, multiplicity of analyses, results from similar studies, and other relevant evidence	n/a
Generalisability	21	Discuss the generalisability (external validity) of the study results	n/a
**Other information**			
Funding	22	Give the source of funding and the role of the funders for the present study and, if applicable, for the original study on which the present article is based	18

*Give information separately for exposed and unexposed groups.

Note: An Explanation and Elaboration article discusses each checklist item and gives methodological background and published examples of transparent reporting. The STROBE checklist is best used in conjunction with this article (freely available on the Web sites of PLoS Medicine at http://www.plosmedicine.org/, Annals of Internal Medicine at http://www.annals.org/, and Epidemiology at http://www.epidem.com/). Information on the STROBE Initiative is available at http://www.strobe-statement.org.

For peer review only

For peer review only - http://bmjopen.bmj.com/site/about/guidelines.xhtml

BMJ Open

Cohort profile: A prospective Australian cohort study of women's reproductive characteristics and risk of chronic disease from menarche to premenopause (M-PreM)

Journal:	*BMJ Open*
Manuscript ID	bmjopen-2022-064333.R1
Article Type:	Cohort profile
Date Submitted by the Author:	08-Sep-2022
Complete List of Authors:	Chan, Hsiu-Wen; The University of Queensland, School of Public Health<br>Dharmage, Shyamali; The University of Melbourne, School of Population and Global Health<br>Dobson, A; The University of Queensland, School of Public Health<br>Chung, Hsin-Fang; The University of Queensland, School of Public Health<br>Loxton, Deborah; The University of Newcastle, Centre for Women's Health Research<br>Doust, Jenny; The University of Queensland, School of Public Health<br>Montgomery, Grant ; The University of Queensland Institute for Molecular Bioscience<br>Stamatakis, Emmanuel; The University of Sydney, Charles Perkins Centre<br>Huxley, Rachel; Deakin University, Faculty of Health; University of New South Wales, The George Institute for Global Health<br>Hamer, Mark; University College London, Epidemiology and public health<br>Abbott, Jason ; University of New South Wales, School of Women's and Children's Health; Royal Hospital for Women, Gynaecological Research and Clinical Evaluation (GRACE) Unit<br>Yeap, Bu; The University of Western Australia, Medical School; Fiona Stanley Hospital, Department of Endocrinology and Diabetes<br>Visser, Jenny; Erasmus MC, University Medical Center<br>McIntyre, Harold; The University of Queensland, School of Medicine; Mater Misericordiae Health Services Brisbane Ltd<br>Mielke , Gregore ; The University of Queensland, School of Public Health<br>Mishra, Gita; The University of Queensland, School of Public Health
**Primary Subject Heading**:	Public health
Secondary Subject Heading:	Epidemiology
Keywords:	PUBLIC HEALTH, Hypertension < CARDIOLOGY, EPIDEMIOLOGY, Asthma < THORACIC MEDICINE

I, the Submitting Author has the right to grant and does grant on behalf of all authors of the Work (as defined in the below author licence), an exclusive licence and/or a non-exclusive licence for contributions from authors who are: i) UK Crown employees; ii) where BMJ has agreed a CC-BY licence shall apply, and/or iii) in accordance with the terms applicable for US Federal Government officers or employees acting as part of their official duties; on a worldwide, perpetual, irrevocable, royalty-free basis to BMJ Publishing Group Ltd ("BMJ") its licensees and where the relevant Journal is co-owned by BMJ to the co-owners of the Journal, to publish the Work in this journal and any other BMJ products and to exploit all rights, as set out in our licence.

The Submitting Author accepts and understands that any supply made under these terms is made by BMJ to the Submitting Author unless you are acting as an employee on behalf of your employer or a postgraduate student of an affiliated institution which is paying any applicable article publishing charge ("APC") for Open Access articles. Where the Submitting Author wishes to make the Work available on an Open Access basis (and intends to pay the relevant APC), the terms of reuse of such Open Access shall be governed by a Creative Commons licence – details of these licences and which Creative Commons licence will apply to this Work are set out in our licence referred to above.

Other than as permitted in any relevant BMJ Author's Self Archiving Policies, I confirm this Work has not been accepted for publication elsewhere, is not being considered for publication elsewhere and does not duplicate material already published. I confirm all authors consent to publication of this Work and authorise the granting of this licence.

For peer review only

Title

Cohort profile: A prospective Australian cohort study of women's reproductive characteristics and risk of chronic disease from menarche to premenopause (M-PreM)

Authors

Hsiu-Wen Chan[1], Shyamali C Dharmage[2], Annette J Dobson[1], Hsin-Fang Chung[1], Deborah Loxton[3], Jenny Doust[1], Grant W Montgomery[4], Emmanuel Stamatakis[5], Rachel R Huxley[6,7], Mark Hamer[8], Jason Abbott[9,10], Bu B Yeap[11,12], Jenny A Visser[13], Harold D McIntyre[14,15], Gregore I Mielke[1], Gita D Mishra[1]

Author affiliations

[1]School of Public Health, The University of Queensland, Brisbane, Queensland, Australia

[2]Allergy and Lung Health Unit, Melbourne School of Population and Global Health, The University of Melbourne, Carlton, Victoria, Australia

[3]Centre for Women's Health Research, School of Medicine and Public Health, University of Newcastle, Newcastle, New South Wales, Australia

[4]The Institute for Molecular Bioscience, The University of Queensland, Brisbane, Queensland, Australia

[5]Charles Perkins Centre, The University of Sydney, Sydney, New South Wales, Australia

[6]Faculty of Health, Deakin University, Burwood, Victoria, Australia

[7]The George Institute for Global Health, University of New South Wales, Sydney, New South Wales, Australia

[8]Division of Surgery and Interventional Sciences, Faculty of Medical Sciences, University College London, United Kingdom

[9]School of Women's and Children's Health, University of New South Wales, Sydney, New South Wales, Australia

[10]Gynaecological Research and Clinical Evaluation (GRACE) Unit, Royal Hospital for Women, Sydney, New South Wales, Australia

[11]Medical School, University of Western Australia, Perth, Western Australia, Australia

[12]Department of Endocrinology and Diabetes, Fiona Stanley Hospital, Perth, Western Australia, Australia

For peer review only

For peer review only - http://bmjopen.bmj.com/site/about/guidelines.xhtml

[13]Department of Internal Medicine, Erasmus MC, University Medical Center Rotterdam, Rotterdam, The Netherlands

[14]School of Medicine, University of Queensland, Brisbane, Queensland, Australia

[15]Mater Research, University of Queensland, Brisbane, Queensland, Australia

Corresponding author

Professor Gita Mishra

The University of Queensland,

School of Public Health

288 Herston Road

Herston QLD 4006

Australia

g.mishra@uq.edu.au

Word count: 3487 words

For peer review only

For peer review only - http://bmjopen.bmj.com/site/about/guidelines.xhtml

ABSTRACT

Purpose

Previous studies have identified associations between individual reproductive factors and chronic disease risk among postmenopausal women. However, few have investigated the association of different markers of reproductive function, their interactions, and risk factors of chronic disease among women approaching menopause. The Menarche-to-PreMenopause (M-PreM) study aims to examine the relationship between reproductive factors across the reproductive lifespan and risk indicators for chronic disease among women in their early-to-mid 40s. The purpose of this cohort profile paper is to describe the rationale, study design, and participant characteristics of the M-PreM study.

Participants

Women born in 1973-78 who participated in the Australian Longitudinal Study on Women's Health (ALSWH) were invited to undertake a clinical or self-administered assessment. A total of 1278 women were recruited from June 2019 to June 2021.

Findings to date

The study measures included functional, cognitive, and cardiometabolic tests, anthropometry, spirometry, respiratory health questionnaires,physical activity, sleep patterns, sex hormones, and cardiovascular and metabolic markers, whereas blood and saliva samples were used for the analysis of genetic variants of genes associated with reproductive characteristics and chronic disease. The mean age of the clinic and self-assessed participants was 44.6 and 45.3 years, respectively. The menopausal status of participants was similar between the two arms of the study: 38-41% pre-menopausal, 20% peri-menopausal, and 36% took oral contraception or hormone replacement therapy. Approximately 80% of women had at least one child and participants reported experiencing pregnancy complications: preterm birth (8-13% of pregnancies), gestational diabetes (10%), and gestational hypertension (10-15%).

Future plans

The biomedical data collected in the M-PreM study will be linked to existing ALSWH survey data on sociodemographic factors, health behaviour, reproductive function, and early life factors collected over the

For peer review only - http://bmjopen.bmj.com/site/about/guidelines.xhtml

past 20 years and health administrative data. The association between reproductive factors and risk indicators of chronic disease will be analysed.

KEYWORDS

Chronic disease, reproduction, women's health

STRENGTHS AND LIMITATIONS OF THIS STUDY

- Biomedical data from the Menarche-to-PreMenopause (M-PreM) study will be linked to two decades of comprehensive survey data and administrative health service records.
- A biobank of blood and saliva samples will enable the investigation of genetic factors that contribute to chronic disease risk.
- The COVID-19 pandemic adversely impacted participation rates for this study and respiratory data collection.
- Due to the complex blood processing requirements, we were ony able to establish study sites in major cities.

INTRODUCTION

Rapidly ageing populations in Australia and globally and the rising tide of associated chronic diseases present a pressing challenge for public health policy. Many age-related chronic diseases show marked sex differences in their prevalence and aetiology, with women being at a greater disadvantage overall. For example, although middle-aged women have a lower prevalence of diabetes[1], women with diabetes are at greater risk of coronary heart disease[2], stroke[3], and all-cause mortality[2] than men with diabetes. After adolescence, the prevalence of asthma is not only higher for Australian women than men across adulthood, but increases rapidly leading up to and through the menopausal transition[4]. Lastly, women have a higher prevalence of non-amnestic mild cognitive impairment than men[5] and experience faster cognitive decline[6].

There is a growing body of evidence on the links between reproductive factors and risk of chronic disease. Large pooled studies have shown that early menarche, early menopause, and a short reproductive lifespan are associated with increased risk of cardiovascular disease in postmenopausal women[7, 8].

For peer review only - http://bmjopen.bmj.com/site/about/guidelines.xhtml

Similarly, meta-analyses and systematic reviews demonstrate associations between early menarche, early menopause, and increased odds of type 2 diabetes[9, 10]. Early menarche is also linked to asthma in early-to-mid adulthood[11].

Few studies have investigated the associations between reproductive health with risk factors of chronic disease in younger women. Early menarche and longer menstrual cycle length (≥33 days) are associated with elevated diastolic blood pressure, total cholesterol, LDL cholesterol, and triglyceride levels among women in their 30s and also those approaching menopause, which may be partially mediated by body composition[12, 13]. Sex hormonal disorders in women of reproductive age, such as polycystic ovary syndrome (PCOS) and premature ovarian insufficiency, have also been linked to cardiometabolic risk factors and/or poor lung function and asthma. Women with PCOS not only have a higher body mass index[14] and waist-to-hip ratio than other women, but they also have higher levels of total and free testosterone and dehydroepiandrosterone (DHEA)[15]. In women with PCOS, there is some evidence that free testosterone is negatively associated with HDL cholesterol levels, whereas DHEA is positively associated with triglyceride and LDL concentration[16]. Sex hormones are also associated with lung function. DHEA is negatively associated with forced expiratory volume in 1 second (FEV1) and forced vital capacity (FVC) and with greater risk of airflow limitation later in life[17]. Other reproductive factors, including early menarche and parity, are associated with poorer lung function (lower FEV1, FVC, and FEV1/FVC; FEV1/FVC is a marker of airway obstruction) and asthma[11, 18, 19].

It has been long recognised that women are at higher risk of developing chronic diseases after menopause, especially cardiometabolic and respiratory disease. However, it is unclear whether reproductive history from adolescence and early-to-mid adulthood affects the risk factors for chronic diseases in the years preceding menopause. Therefore, in order to implement targeted early preventative health strategies, there is a pressing need to examine the relationship between reproductive factors, sex hormones, and risk factors of chronic disease in women before they enter the menopausal transition. Additionally, whilst previous studies have identified sex-specific risk factors for chronic conditions, many have viewed reproductive characteristics in isolation, rather than as a series of related and interacting factors.

The Menarche-to-PreMenopause (M-PreM) study aims to:

For peer review only

1) investigate links between reproductive factors and markers of cardiometabolic health, respiratory, and cognitive and functional health among women before they reach middle-age, and
2) determine whether body size (anthropometry) and physical activity modify these relationships.

The markers of cardiometabolic health measured are glycated haemoglobin (HbA1c), serum lipids, and blood pressure and the markers of respiratory health will be obtained from lung function tests and questionnaires. Other measures include functional and cognitive tests. In addition, this study will use existing data on physical and mental health, reproductive factors and sociodemographic data from the Australian Longitudinal Study on Women's Health surveys and linked administrative health records. This paper describes the recruitment of participants, data collection protocol, and participant characteristics of the M-PreM study.

COHORT DESCRIPTION

The Australian Longitudinal Study on Women's Health (ALSWH) is a national, prospective study of women's biological and mental health, social and lifestyle factors, and health service use. In 1996, women born in 1921-26, 1945-51, and 1973-78 were randomly selected from the database of Medicare (Australia's universal health insurance scheme) with intentional over-sampling of women from rural and remote areas. Data collection of this currently ongoing study primarily comprises self-report surveys conducted approximately every three years. More information about the ALSWH can be found elsewhere[20, 21].

Exclusion and inclusion criteria

The 8th survey of the 1973-1978 ALSWH cohort was conducted in 2018 when the participants were aged 40-45 years and completed by 7121 participants. In this survey, women were introduced to the M-PreM study and asked to indicate their interest in participating. Those who responded positively (n=4584) were invited to the M-PreM study (Figure 1). The exclusion criteria included women who were pregnant or had been diagnosed with a breast or reproductive cancer and were undergoing treatment at the time of invitation. Chemotherapy and radiation treatments for breast or reproductive cancers often affect reproductive function and blood sex hormone concentration[22].

Sample size

For peer review only - http://bmjopen.bmj.com/site/about/guidelines.xhtml

For peer review only

The M-PreM study commenced in June 2019 and concluded in June 2021. Due to the SARS-CoV-2 (COVID-19) outbreak in Australia, the study was suspended from March 2020 to November 2020. The operations of this study and participation have been adversely affected by sudden periods of lockdown in different Australian states, border closures, and new health and safety regulations for clinical research. Overall, 1278 participants were recruited: 499 participants had completed a clinical assessment and 779 participants completed a self-administered assessment (Figure 1).

Based on conservative estimates, sample sizes of 499 and 779 for the clinical and self-administered assessments, respectively, provide enough power to detect associations between exposure-outcome (e.g. the association between early menarche and blood pressure) of small to medium magnitudes after adjustment for up to 10 variables using linear regression models. Assuming a power of 80%, a significance level of 5% and a model including covariates that explains 10% of the variation in the outcome (reduced model; R^2 of 0.10), the minimum detectable values for the R^2 difference between the reduced and full models (model including 10 covariates and the exposure of interest) are 0.014 (R^2 of the full model 0.114) and 0.009 (R^2 of the full model: 0.109) for the clinical and self-administered assessments, respectively. For a power of 90%, the minimum detectable values for the R^2 difference between the reduced and full models are 0.019 for the clinical assessment and 0.012 for the self-administered assessment.

DATA COLLECTION AND MEASURES

The M-PreM study will analyse existing longitudinal and linked data from the ALSWH in combination with new biomedical data.

Existing data from the ALSWH

The 1973-78 cohort have completed up to 9 surveys approximately every 3 years since 1996 (the 9th survey was conducted in 2021-2022; Figure 2). From these surveys, data on socio-demographic factors, self-reported anthropometry, health-related behaviours, and reproductive function are available. The specific items relevant for the M-PreM study are detailed in Table 1.

Table 1 Self-reported data from the ALSWH relevant for the M-PreM study

Category	Item*
Sociodemographic factors	Highest educational attainment<br>Area of residence

For peer review only - http://bmjopen.bmj.com/site/about/guidelines.xhtml

	Marital status
Anthropometry (self-reported)	Weight<br>Height<br>Waist circumference
Health-related behaviour	Current and lifetime smoking history<br>Alcohol intake (quantity and frequency)<br>Use of illicit drugs<br>Level of physical activity<br>Sedentary behaviour, self-reported sitting time<br>Dietary intake, food frequency questionnaires
Reproductive function	Age at menarche<br>Dysmenorrhoea<br>Irregular periods<br>Premenstrual syndrome<br>Age at first delivery<br>Number of miscarriages<br>Terminations<br>Stillbirths<br>Live births<br>Diagnosis of endometriosis or polycystic ovary syndrome<br>Use of fertility treatment<br>Use of contraception<br>Hysterectomy and oophorectomy<br>Diagnosed with gestational diabetes or gestational hypertension<br>Premature birth<br>Infant birthweight<br>Duration of breastfeeding
Early life factors	Early life nutrition (breastfeeding) (Adapted from the Longitudinal Study of Australian Children)<br>Mother's pregnancy complications<br>Attitude and behaviour of parents/caregivers during childhood [23]<br>Childhood household condition [24]<br>Childhood health [25]

*Information for the standard questions administered in the ALSWH (not referenced in this table) can be found here[20, 21].

Modes of participation

Study sites were established in the Translational Research Institute (Brisbane), Murdoch Children's Research Institute (Melbourne), Royal Hospital for Women (Sydney), PARC Clinical Research (Adelaide), and Harry Perkins Institute of Medical Research (Perth). Participants were invited to attend a clinical assessment where they undertook a range of biomedical measurements, provided a non-fasting blood sample, were fitted with a physical activity monitor which they wore for 8 days, and completed an 8-day sleep diary (Table 2). Participants who reported 'ever having PCOS' were also asked to complete a 3-month menstrual diary. Before the COVID-19 pandemic, participants conducted pre- and post-bronchodilator spirometry, however this was replaced by a respiratory health questionnaire after the outbreak emerged for health and safety reasons (more details can be found in the spirometry methods section). Where participants were unable to

For peer review only - http://bmjopen.bmj.com/site/about/guidelines.xhtml

attend a study site, they were sent a package that allowed collection of self-administered data. This contained an Oragene·DNA saliva collection kit (OG-600; DNA Genotek Inc.), and an accelerometer (Table 2). The package was then returned via Reply Paid post to the study team at the University of Newcastle.

Table 2 Questionnaires, measurements, biological samples collected and biological markers measured.

**Category**	**Clinic**	**Self-administered**
Reproductive characteristics		
Contraception use [20]	✓	✓
Hormone therapy use [20]	✓	✓
Menopausal status [20]	✓	✓
Ever had PCOS	✓	✓
Menstrual cycle staging [26]	✓	✓
3-month menstrual diary (if ever had PCOS)	✓	✓
Respiratory health* (before 13/03/2020)		
Attacks of asthma or wheezy breathing [27, 28]	✓	✓
Use of medications that affect the airways	✓	
Recent respiratory illness	✓	
Spirometry	✓	
Respiratory health* (after 13/03/2020)		
Attacks of asthma or wheezy breathing [27, 28]	✓	✓
Attacks of cough [27, 28]	✓	✓
Phlegm in the chest [27, 28]	✓	✓
History of pneumonia and other chest illnesses [27, 28]	✓	✓
Ever had bronchitis, COPD, and emphysema	✓	✓
COVID-19	✓	✓
Cognition		
Montreal Cognitive Assessment [29]	✓	
Functional tests		
Handgrip strength test [30]	✓	
Chair rise test [31]	✓	
Standing balance test [31]	✓	
Anthropometry		
Height	✓	✓
Weight	✓	✓
Body mass index	✓	✓
Waist circumference	✓	✓
Hip circumference	✓	✓
Arm circumference	✓	✓
Thigh circumference	✓	✓
Physical activity		
ActivPAL accelerometers worn for 8 days [32]	✓	✓
Sleep patterns		
8-day sleep diary[32]	✓	✓
Biological samples		
Whole blood	✓	
Serum	✓	
Plasma	✓	
Buffy coat	✓	
Saliva		✓
Cardiometabolic tests		
Blood pressure	✓	

For peer review only

Resting heart rate	✓	
Total cholesterol	✓	
HDL cholesterol	✓	
LDL cholesterol	✓	
Glycated haemoglobin (HbA1c)	✓	
Sex hormones		
Estradiol (E2)	✓	
Estrone (E1)	✓	
Testosterone	✓	
Dihydrotestosterone (DHT)	✓	
Progesterone (P)	✓	
Dehydroepiandrosterone (DHEA)	✓	
Anti-Mullerian Hormone (AMH)	✓	
Sex hormone-binding globulin (SHBG)	✓	
Follicle-stimulating hormone (FSH)	✓	
Luteinising hormone (LH)	✓	

PCOS, polycystic ovary syndrome; COPD, chronic obstructive pulmonary disorder.
*The respiratory health questionnaire and measurements changed after the onset of the COVID-19 pandemic.

Questionnaires

Reproductive characteristics, including use of contraception and hormone therapy, menopausal status, and self-reported polycystic ovary syndrome (PCOS), have been captured using questions from the ALSWH surveys[20]. Menstrual cycle stage at the time of assessment was determined using questions from the Seattle Midlife Women's Health Study[26]. Respiratory health, including attacks of asthma or wheezy breathing, whistling in the chest, shortness of breath and chest tightness was determined using the questionnaire from the Follow-up of the Tasmanian Longitudinal Health Study from first to sixth decade[27], which was adapted from the European Community Respiratory Health Survey II [28]. Cognition was assessed using the Montreal Cognitive Assessment[29].

Handgrip strength test

Handgrip strength was measured (in kilograms) using Jamar hydraulic hand dynamometer (model 5030J1)[30]. Participants were seated with a straight-back posture and their feet flat on the ground. They were asked to hold their elbow against their side at a 90° angle and their forearm and wrist in a neutral position. The handgrip strength test was performed once in each hand.

For peer review only

For peer review only - http://bmjopen.bmj.com/site/about/guidelines.xhtml

Chair rise test

After removing their shoes, participants were asked to sit in a straight-backed armless chair with a horizontal, flat seat. Participants were assessed on the length of time (in seconds) taken to complete 10 sets of a sit-stand-sit manoeuvre[31].

Standing balance test

Participants were tested for the length of time (in seconds) that they could maintain a one-legged stance for a maximum of 30 seconds, firstly with their eyes open and then closed[31]. This test was performed without shoes and with both arms crossed over the chest. Participants were asked to stand on their preferred foot and to raise their non-preferred foot off the floor by bending their knee.

Blood pressure and heart rate at rest

After resting in a seated position for 5 minutes, three measurements of systolic and diastolic blood pressures and heart rate, interspersed by 1-2 minutes breaks, was recorded using an automated blood pressure monitor. For analysis, only the second and third measurements were averaged, as recommended by the World Health Organization protocol[33].

Anthropometric assessment

Weight without shoes was measured once using a digital scale to the nearest 0.1kg. Height without shoes was measured once using a stadiometer to the nearest millimetre. Using these measurements, body mass index (kg/m^2) was calculated. The waist circumference was measured at the midpoint between the bottom of the last palpable rib and top of the iliac crest (for self-assessment, this was described as ‘the midpoint between bottom of the ribs and top of the hip’). The hip circumference was measured at the widest point of the buttocks (for self-assessment, this was described as the ‘widest portion of the bottom’). The arm circumference was measured at the midpoint between the acromion process of the scapula and the olecranon process (for self-assessment, this was described as the ‘midpoint between the top of your shoulder to the point of your elbow’). Thigh circumference was measured at the midpoint between the lateral superior margin of the patella and the anterior superior iliac spine (for self-assessment, this was described as the ‘midpoint between the top of your hip to the top of your knee’). Circumference measurements in the clinic were conducted to the nearest millimetre in duplicate, whereas at home, participants were asked to record the

For peer review only - http://bmjopen.bmj.com/site/about/guidelines.xhtml

measurements in centimetres to one decimal point. Where the difference between the two measurements exceeded 5mm, a third measurement was performed. The average of the two or three measurements was then calculated.

Spirometry

Lung function testing was conducted from 9 July 2019 to 13 March 2020, after which it was removed from the protocol due to health and safety concerns as a result of the COVID-19 pandemic. Lung function testing using Easy-on-PC spirometers (NDD Medizintechnik AG, Switzerland) was performed according to the American Thoracic Society and European Respiratory Society guidelines[34] and based on the protocols of the Tasmanian Longitudinal Health Study[27]. Participants were required to perform a minimum of three acceptable trials for each pre- and post-bronchodilator test. Post-bronchodilator spirometry was performed 10-15 minutes after the bronchodilator, salbutamol (300µg), was administered via a spacer.

To replace the lung function testing in the context of the COVID-19 pandemic, an extended respiratory health questionnaire was then given to all participants who were recruited after the M-PreM study resumed on 10 September 2020. In addition to the original respiratory health questions (detailed in the Questionnaires section), the following questions were asked: ever had and age of diagnosis of bronchitis, chronic obstructive pulmonary disorder, and emphysema; and history of bronchitis, phlegm in the chest, cough in the absence of a cold, cough with phlegm, and chest illnesses including pneumonia. Like the original respiratory health questions, these questions were also sourced from the Follow-up of the Tasmanian Longitudinal Health Study from first to sixth decade[27]. Lastly, questions about COVID-19 testing and results as well as self-reported changes to participant's respiratory function were in the extended respiratory health questionnaire.

Participants who had already completed a clinical or self-administered assessment prior to the onset of the COVID-19 pandemic were also invited to complete the extended respiratory health questionnaire.

Physical activity and sleep patterns

Accelerometers (activPAL3 micro and activPAL4 micro devices; PAL Technologies, UK) were attached to the middle of the anterior surface of the right thigh using adhesive dressing (3M Tegaderm) and worn continuously for 8 full days. Participants were asked to log their sleep patterns (bedtime, estimated time they

For peer review only - http://bmjopen.bmj.com/site/about/guidelines.xhtml

fell asleep, waking time, and time they got out of bed) and sleep quality (number of night wakings, how well they slept) for 8 days.

Biological samples and biomarker testing

BD Vacutainer® EDTA (for plasma and HbA1c testing), SST II (for serum and cholesterol testing), and plain tubes (for serum) were used to collect non-fasting venous blood. Whole blood, serum, plasma and buffy coat were also extracted. All samples are biobanked at -80°C at the Institute of Molecular Biosciences (Brisbane, Australia).

Serum estrone (E1), estradiol (E2), testosterone, dihydrotestosterone (DHT), progesterone (P), and dehydroepiandrosterone (DHEA) concentrations were analysed using a validated liquid chromatography-mass spectrometry (LC-MS) method by the Andrology Laboratory at the ANZAC Research Institute (Sydney, Australia)[35, 36]. Serum sex hormone-binding globulin (SHBG), follicle-stimulated hormone (FSH), and luteinizing hormone (LH) concentrations were analysed by the Concord Repatriation General Hospital Diagnostic Pathology Unit (Sydney, Australia) using commercial immunoassays (Roche). Anti-Mullerian Hormone concentration in serum will be measured by NSW Health Pathology (Sydney, Australia). Total, LDL-, and HDL-cholesterol and glycated haemoglobin A1c (HbA1c) was measured by Pathology Queensland (Central Laboratory, Brisbane, Australia) using routine auto-analyser methods. In the future, DNA will be extracted from the stored blood and saliva samples to identify genetic factors associated with reproductive factors, and cardiometabolic and respiratory conditions. The differences in samples should not affect DNA sequences, but could possibly affect epigenetic markers.

Patient and public involvement

A pilot study was conducted to develop and test the study protocol and provided an opportunity for participant feedback.

FINDINGS TO DATE

A total of 1278 participants were recruited to the M-PreM study. Of these, 499 attended a clinical study site and 779 completed a self-assessment. The demographic characteristics of these participants were based on their responses in the 8th survey of the ALSWH (Table 3) and compared with the remaining cohort who did

For peer review only - http://bmjopen.bmj.com/site/about/guidelines.xhtml
For peer review only

not participate in M-PreM. As expected, those who completed a clinical assessment predominantly lived in a major city whereas those who participated in the self-assessment were spread amongst major cities, and inner and outer regional areas. Compared with the participants who completed the self-assessment or did not participate in the M-PreM study, in-clinic participants were more likely to be university-educated and less likely to have 3 or more children, to ever have smoked or have a history of preterm birth. They also started having children at a later age. Participants in the M-PreM study, regardless of mode of participation, were more likely to report better health and be non-drinkers than non-participants.

Table 3 Characteristics of the M-PreM participants and the remainder of the ALSWH 1973-78 cohort.

	M-PreM Clinical assessment n=499 (7.01%)	M-PreM Self-assessment n=779 (10.94%)	1973-78 ALSWH Survey 8 cohort, not in M-PreM n=5843 (82.05%)	p-value*
Area of residence, n (%)				
Major city	463 (93.16)	313 (40.44)	3248 (57.40)	<0.0001
Inner regional	26 (5.23)	275 (35.53)	1594 (28.17)	
Outer regional	4 (0.80)	164 (21.19)	695 (12.28)	
Remote	4 (0.80)	22 (2.84)	122 (2.16)	
Missing	2	5	184	
Highest qualification, n (%)				
Less than year 12 or equiv.	10 (2.00)	39 (5.01)	305 (5.55)	<0.0001
Year 12 or equiv.	24 (4.81)	68 (8.73)	529 (9.62)	
Trade/apprenticeship/Cert/Dip	92 (18.44)	229 (29.40)	1586 (28.85)	
University	373 (74.75)	443 (56.87)	3078 (55.98)	
Missing	0	0	345	
Marital status, n (%)				
Never married	58 (11.62)	77 (9.88)	636 (11.55)	0.771
Married/De facto	390 (78.16)	624 (80.10)	4299 (78.08)	
Separated	26 (5.21)	33 (4.24)	236 (4.29)	
Divorced	22 (4.41)	40 (5.13)	309 (5.61)	
Widowed	3 (0.60)	5 (0.64)	26 (0.47)	
Missing	0	0	337	
Employment status, n (%)				
Not in labour force	60 (12.02)	103 (13.24)	840 (14.89)	0.028
Employed full time	225 (45.09)	330 (42.42)	2529 (44.82)	
Employed part time	180 (36.07)	274 (35.22)	1752 (31.05)	
Employed casually	34 (6.81)	71 (9.13)	521 (9.23)	
Missing	0	1	201	
Self-reported health, n (%)				
Excellent/very good	300 (60.12)	477 (61.23)	3130 (53.89)	0.0002
Good	157 (31.46)	220 (28.24)	2003 (34.49)	
Fair/poor	42 (8.42)	82 (10.53)	675 (11.62)	
Missing	0	0	35	
Smoking status, n (%)				
Never smoked	344 (68.94)	482 (61.95)	3428 (61.00)	0.0002
Ex-smoker	125 (25.05)	235 (30.21)	1587 (28.24)	
Current smoker	30 (6.01)	61 (7.84)	605 (10.77)	
Missing	0	1	223	
Alcohol use, n (%)				
Non-drinker	39 (7.82)	64 (8.23)	609 (10.82)	0.014
Low risk	432 (86.57)	663 (85.22)	4598 (81.73)	

For peer review only - http://bmjopen.bmj.com/site/about/guidelines.xhtml

High risk	28 (5.61)	51 (6.56)	419 (7.45)	
Missing	0	1	217	
Number of children, n (%)				
No children	105 (21.04)	127 (16.30)	1149 (19.66)	0.005
1 child	56 (11.22)	96 (12.32)	789 (13.50)	
2 children	229 (45.89)	345 (44.29)	2328 (39.84)	
3 or more children	109 (21.84)	211 (27.09)	1577 (26.99)	
Missing	0	0	0	
Age at menarche, n (%)				
11 years or younger	52 (12.53)	74 (11.23)	551 (11.51)	0.749
12 years	115 (27.71)	185 (28.07)	1371 (28.63)	
13 years	124 (29.88)	202 (30.65)	1482 (30.95)	
14 years	80 (19.28)	106 (16.08)	813 (16.98)	
15 years or older	44 (10.60)	92 (13.96)	571 (11.93)	
Missing	84	120	1055	
History of preterm birth, n (%)				
Yes	33 (8.38)	87 (13.43)	668 (14.49)	0.003
No	361 (91.62)	561 (86.57)	3943 (85.51)	
Missing	105	131	1232	
History of stillbirth, n (%)				
Yes	11 (2.79)	7 (1.07)	92 (1.96)	0.128
No	383 (97.21)	645 (98.93)	4593 (98.04)	
Missing	105	127	1158	
History of gestational diabetes (%)				
Yes	39 (9.90)	64 (9.89)	490 (10.63)	0.782
No	355 (91.10)	583 (90.11)	4119 (89.37)	
Missing	105	132	1234	
History of gestational hypertension (%)				
Yes	39 (9.92)	98 (15.15)	646 (14.02)	0.0476
No	354 (90.08)	549 (84.85)	3963 (85.98)	
Missing	106	132	1234	
Age at first birth, mean (SD)	31.31 (4.92)	29.67 (5.16)	29.59 (5.28)	<0.0001

*Analysis was conducted using ANOVA and Chi-square tests.

Table 4 shows some of the outcome measurements. At the time of participation, approximately 40% of the women were premenopausal and 20% had begun their menopausal transition, regardless of their mode of participation. Over 55% of participants were categorised as overweight or obese based on their body mass index (BMI). The BMI and body measurements were similar between the clinic- and self-assessed participants, except arm and thigh circumference, which were larger in clinic participants. Also, the prevalence of respiratory symptoms was similar between the clinic- and self-assessed participants.

Table 4 Clinical outcomes from the Menarche-to-PreMenopause study.

	Clinic assessment (n=499)		Self-assessment (n=779)		p-value
	n		n		
Age (years), mean (SD)	499	44.60 (1.61)	779	45.28 (1.78)	<0.001
Menopausal status, %					
Surgical menopause	8	1.61	20	2.73	0.531
Not defined due to HRT or OCP use	177	35.61	263	35.93	

For peer review only - http://bmjopen.bmj.com/site/about/guidelines.xhtml

Pre-menopausal	202	40.64	275	37.57	
Peri-menopausal	99	19.92	152	20.77	
Post-menopausal	11	2.21	22	3.01	
Missing	2		47		
Body mass index category, %					
Underweight <18.5kg/m²)	2	0.40	8	1.10	0.380
Normal weight (18.5 to <25kg/m²)	202	40.56	311	42.60	
Overweight (25 to <30kg/m²)	147	29.52	193	26.44	
Obese (≥30kg/m²)	147	29.52	218	29.86	
Missing	1		49		
Body mass index, mean (SD)	498	27.80 (6.43)	730	27.68 (6.48)	0.750
Body measurements (cm), mean (SD)					
Waist circumference	499	88.78 (14.84)	748	89.18 (14.98)	0.647
Hip circumference	498	106.21 (13.49)	749	105.97 (13.62)	0.755
Arm circumference	498	31.27 (5.13)	736	30.36 (4.35)	0.001
Thigh circumference	498	59.06 (8.47)	746	55.74 (7.78)	<0.001
Blood Pressure (mmHg), mean (SD)					
Systolic	494	119.11 (12.37)	-	-	n/a
Diastolic	494	77.29 (8.84)	-	-	
Resting heart rate (beats per min), mean (SD)	493	75.15 (10.50)	-	-	n/a
Cardiometabolic biomarkers, mean (SD)					
Total cholesterol (mmol/L)	489	5.21 (0.89)	-	-	n/a
HDL cholesterol (mmol/L)	489	1.64 (0.39)	-	-	
LDL cholesterol (mmol/L)	486	3.00 (0.77)	-	-	
HbA1c (%)	489	5.14 (0.36)	-	-	
Handgrip strength (kg), mean (SD)					n/a
Dominant hand	486	30.57 (6.01)	-	-	
Non-dominant hand	486	28.62 (5.26)	-	-	
Standing balance test (seconds)*, mean (SD)					n/a
Eyes open	498	28.57 (5.26)	-	-	
Eyes closed	498	10.97 (10.36)	-	-	
Chair rise test (seconds), mean (SD)	495	16.72 (4.77)	-	-	n/a
Reciprocal of the chair rise test**, mean (SD)	495	6.48 (1.86)			
Montreal Cognitive Assessment***, mean (SD)	499	27.69 (1.94)	-	-	n/a
Lung function, prebronchodilator, mean (SD)					
FEV1 (L)	283	3.03 (0.45)	-	-	n/a
FVC (L)	283	3.84 (0.56)	-	-	
FEV1/FVC (%)	283	0.79 (0.05)	-	-	
Lung function, postbronchodilator, mean (SD)					
FEV1 (L)	283	3.12 (0.46)	-	-	n/a
FVC (L)	283	3.84 (0.56)	-	-	
FEV1/FVC (%)	283	0.82 (0.05)	-	-	
Ever had asthma or wheezy breathing (%)					
Yes	194	38.96	279	36.23	0.328
No	304	61.04	491	63.77	
Missing	1		9		
Had asthma or wheezy breathing in the last 12 months (%)					
Yes	82	42.27	139	49.12	0.141
No	112	57.73	144	50.88	
Missing	305		496		
Wheeze or whistling in your chest in the last 12 months (%)					
Yes	79	15.86	137	17.77	0.378
No	419	84.14	634	82.23	
Missing	1		8		
Ever had attacks of cough with phlegm (%)					
Yes	239	51.84	361	51.87	0.994
No	222	48.16	335	48.13	

For peer review only

Missing	38		83		
Ever had cough in the absence of a cold (%)					
Yes	116	25.16	141	20.17	0.045
No	345	74.84	558	79.83	
Missing	38		80		
Ever had phlegm in your chest in the absence of a cold (%)					
Yes	28	6.07	49	7.01	0.531
No	433	93.93	650	92.99	
Missing	38		80		

Analysis was conducted using t-tests and Chi-square tests.
*Maximum score of 30 seconds
** Reciprocal of chair rise time x 100
**Score out of 30

For peer review only

STRENGTHS AND LIMITATIONS

The strength of the M-PreM study is that it presents an invaluable opportunity to investigate the relationship between reproductive characteristics across the reproductive lifespan and risk indicators for chronic disease and functional and mental health before midlife. In spite of the difficult circumstances due to the COVID-19 pandemic, we have succeeded in recruiting a large sample of participants who have completed questionnaires, clinical measures, physical activity assessments, and blood sample collection to augment the existing information available from the ALSWH surveys. The distributions of measurements were well within the expected ranges found in other studies for handgrip strength[37], body mass index[38], systolic and diastolic blood pressure[38, 39], cholesterol[40, 41], waist circumference[38], hip circumference[42, 43], thigh circumference[42, 43], and arm circumference[44]. The mean Montreal Cognitive Assessment scores was higher in our cohort compared with other studies[45, 46], possibly due to the high representation of women with university education in our cohort. In addition, data from this study will provide the baseline for a follow-up of participants in 6 years' time to determine the relationship between reproductive history and women's health before and after menopause (Figure 2).

The conduct of the study was affected by the coronavirus (COVID-19) pandemic. After two lengthy study suspensions, the participation rate was lower than expected as participants were uncertain about attending hospitals where the study sites were established and people continued to work from home and juggle child care responsibilities, even after the government lifted stay-at-home orders. COVID-19 outbreaks occurred sporadically throughout the study period and study sites were either closed at short notice or their establishment was halted. In addition, the study protocol was modified to minimise risk of COVID-19

For peer review only - http://bmjopen.bmj.com/site/about/guidelines.xhtml

transmission between study personnel and participants. Specifically, the objective lung function testing was replaced by a self-reported respiratory health questionnaire. Participation was also affected by the 2019-2020 Australian bushfire disaster, which prevented a study site from being established in Canberra. Another limitation was the location of the study sites. Although ALSWH is a national study, for M-PreM, study sites could only be established in major cities.

Regardless of the limitations, the study will be able to use this wealth of data to make a substantial contribution to understanding female reproductive health.

COLLABORATION

Researchers interested in collaborating on the M-PreM study can contact the ALSWH on info@alswh.org.au or visit www.alswh.org.au for more information about the study, the available data, and the requirements for accessing the data.

FURTHER DETAILS

Ethics approval and consent to participate

The study has been approved by the Metro South Health and Health Services Human Research Ethics Committee (Brisbane, Australia; Ref: HREC/2019/QMS/52052) and ratified by the University of Newcastle and the University of Queensland Human Research Ethics Committees. All participants provided informed consent by completing an electronic or paper participant consent form.

Data availability statement

Data are available upon reasonable request. Access to the M-PreM dataset requires approval from the Australian Longitudinal Study on Women's Health Data Access Committee. More information can be found at the ALSWH website: https://alswh.org.au/for-data-users/.

Funding

This work is supported by the National Health and Medical Research Council Project Grant (APP1129592). GM is supported by an NHMRC Principal Research Fellowship (APP1121844).

For peer review only - http://bmjopen.bmj.com/site/about/guidelines.xhtml

Author contributions

GDM designed the study, is the lead applicant on the funding application. SCD, JAV, DL, RRH, AJD, GWM, HDM, and JD are co-applicants of the funding application and provided feedback on the design and the operations of the study. SCD, JA, and BBY were study site principal investigators and provided on-site supervision. ES and MH provided accelerometers and protocols for physical activity data collection. H-WC is the project coordinator for the study and drafted this manuscript. H-FC is the postdoctoral fellow for this study and provided feedback on the study operations. GIM is a postdoctoral fellow for this study and provided statistical expertise. All authors reviewed and approved the final manuscript.

Acknowledgements

We would like to acknowledge the Australian Government Department of Health for funding the Australian Longitudinal Study on Women's Health and the women who provided survey data and biomedical data for this study.

Consent for publication

Not applicable

Competing interests

The authors declare no competing interests.

LICENCE STATEMENT

I, the Submitting Author has the right to grant and does grant on behalf of all authors of the Work (as defined in the below author licence), an exclusive licence and/or a non-exclusive licence for contributions from authors who are: i) UK Crown employees; ii) where BMJ has agreed a CC-BY licence shall apply, and/or iii) in accordance with the terms applicable for US Federal Government officers or employees acting as part of their official duties; on a worldwide, perpetual, irrevocable, royalty-free basis to BMJ Publishing Group Ltd ("BMJ") its licensees and where the relevant Journal is co-owned by BMJ to the co-owners of the Journal, to publish the Work in BMJ Open and any other BMJ products and to exploit all rights, as set out in our licence.

For peer review only - http://bmjopen.bmj.com/site/about/guidelines.xhtml

For peer review only

The Submitting Author accepts and understands that any supply made under these terms is made by BMJ to the Submitting Author unless you are acting as an employee on behalf of your employer or a postgraduate student of an affiliated institution which is paying any applicable article publishing charge ("APC") for Open Access articles. Where the Submitting Author wishes to make the Work available on an Open Access basis (and intends to pay the relevant APC), the terms of reuse of such Open Access shall be governed by a Creative Commons licence – details of these licences and which Creative Commons licence will apply to this Work are set out in our licence referred to above.

REFERENCES

1. Saeedi, P., et al., *Global and regional diabetes prevalence estimates for 2019 and projections for 2030 and 2045: Results from the International Diabetes Federation Diabetes Atlas, 9(th) edition.* Diabetes Res Clin Pract, 2019. **157**: p. 107843.
2. Wang, Y., et al., *Sex differences in the association between diabetes and risk of cardiovascular disease, cancer, and all-cause and cause-specific mortality: a systematic review and meta-analysis of 5,162,654 participants.* BMC Med, 2019. **17**(1): p. 136.
3. Peters, S.A., R.R. Huxley, and M. Woodward, *Diabetes as a risk factor for stroke in women compared with men: a systematic review and meta-analysis of 64 cohorts, including 775,385 individuals and 12,539 strokes.* Lancet, 2014. **383**(9933): p. 1973-80.
4. Leynaert, B., et al., *Gender differences in prevalence, diagnosis and incidence of allergic and non-allergic asthma: a population-based cohort.* Thorax, 2012. **67**(7): p. 625-31.
5. Au, B., S. Dale-McGrath, and M.C. Tierney, *Sex differences in the prevalence and incidence of mild cognitive impairment: A meta-analysis.* Ageing Res Rev, 2017. **35**: p. 176-199.
6. Levine, D.A., et al., *Sex Differences in Cognitive Decline Among US Adults.* JAMA Netw Open, 2021. **4**(2): p. e210169.
7. Zhu, D., et al., *Age at natural menopause and risk of incident cardiovascular disease: a pooled analysis of individual patient data.* Lancet Public Health, 2019. **4**(11): p. e553-e564.
8. Mishra, S.R., et al., *Association Between Reproductive Life Span and Incident Nonfatal Cardiovascular Disease: A Pooled Analysis of Individual Patient Data From 12 Studies.* JAMA Cardiol, 2020. **5**(12): p. 1410-1418.
9. Cheng, T.S., et al., *Association of puberty timing with type 2 diabetes: A systematic review and meta-analysis.* PLoS Med, 2020. **17**(1): p. e1003017.
10. Anagnostis, P., et al., *Early menopause and premature ovarian insufficiency are associated with increased risk of type 2 diabetes: a systematic review and meta-analysis.* Eur J Endocrinol, 2019. **180**(1): p. 41-50.
11. Lieberoth, S., et al., *Age at menarche and risk of asthma: systematic review and meta-analysis.* J Asthma, 2014. **51**(6): p. 559-65.
12. Bubach, S., et al., *Early age at menarche and metabolic cardiovascular risk factors: mediation by body composition in adulthood.* Sci Rep, 2021. **11**(1): p. 148.
13. Matthews, K.A., et al., *Relation of cardiovascular risk factors in women approaching menopause to menstrual cycle characteristics and reproductive hormones in the follicular and luteal phases.* J Clin Endocrinol Metab, 2006. **91**(5): p. 1789-95.
14. Teede, H.J., et al., *Longitudinal weight gain in women identified with polycystic ovary syndrome: results of an observational study in young women.* Obesity (Silver Spring), 2013. **21**(8): p. 1526-32.

For peer review only - http://bmjopen.bmj.com/site/about/guidelines.xhtml

15. Perez-Lopez, F.R., et al., *Circulating kisspeptin and anti-mullerian hormone levels, and insulin resistance in women with polycystic ovary syndrome: A systematic review, meta-analysis, and meta-regression.* Eur J Obstet Gynecol Reprod Biol, 2021. **260**: p. 85-98.
16. Amiri, M., et al., *Relationships Between Biochemical Markers of Hyperandrogenism and Metabolic Parameters in Women with Polycystic Ovary Syndrome: A Systematic Review and Meta-Analysis.* Horm Metab Res, 2019. **51**(1): p. 22-34.
17. Pesce, G., et al., *Low serum DHEA-S is associated with impaired lung function in women.* EClinicalMedicine, 2020. **23**: p. 100389.
18. Tang, R., A. Fraser, and M.C. Magnus, *Female reproductive history in relation to chronic obstructive pulmonary disease and lung function in UK biobank: a prospective population-based cohort study.* BMJ Open, 2019. **9**(10): p. e030318.
19. Campbell, B., et al., *Early menarche is associated with lower adult lung function: A longitudinal cohort study from the first to sixth decade of life.* Respirology, 2020. **25**(3): p. 289-297.
20. Dobson, A.J., et al., *Cohort Profile Update: Australian Longitudinal Study on Women's Health.* Int J Epidemiol, 2015. **44**(5): p. 1547,1547a-1547f.
21. Lee, C., et al., *Cohort Profile: the Australian Longitudinal Study on Women's Health.* Int J Epidemiol, 2005. **34**(5): p. 987-91.
22. Meirow, D. and D. Nugent, *The effects of radiotherapy and chemotherapy on female reproduction.* Hum Reprod Update, 2001. **7**(6): p. 535-43.
23. Steptoe, A., et al., *Cohort profile: the English longitudinal study of ageing.* Int J Epidemiol, 2013. **42**(6): p. 1640-8.
24. Kraal, A.Z., et al., *Physical Activity in Early- and Mid-Adulthood Are Independently Associated With Longitudinal Memory Trajectories in Later Life.* J Gerontol A Biol Sci Med Sci, 2021. **76**(8): p. 1495-1503.
25. Pakpahan, E., R. Hoffmann, and H. Kroger, *Retrospective life course data from European countries on how early life experiences determine health in old age and possible mid-life mediators.* Data Brief, 2017. **10**: p. 277-282.
26. Mitchell, E.S., N.F. Woods, and A. Mariella, *Three stages of the menopausal transition from the Seattle Midlife Women's Health Study: toward a more precise definition.* Menopause, 2000. **7**(5): p. 334-49.
27. Tan, D.J., et al., *Clinical and functional differences between early-onset and late-onset adult asthma: a population-based Tasmanian Longitudinal Health Study.* Thorax, 2016. **71**(11): p. 981-987.
28. European Community Respiratory Health Survey, I.I.S.C., *The European Community Respiratory Health Survey II.* Eur Respir J, 2002. **20**(5): p. 1071-9.
29. Nasreddine, Z.S., et al., *The Montreal Cognitive Assessment, MoCA: a brief screening tool for mild cognitive impairment.* J Am Geriatr Soc, 2005. **53**(4): p. 695-9.
30. Spruit, M.A., et al., *New normative values for handgrip strength: results from the UK Biobank.* J Am Med Dir Assoc, 2013. **14**(10): p. 775 e5-11.
31. Kuh, D., et al., *Grip strength, postural control, and functional leg power in a representative cohort of British men and women: associations with physical activity, health status, and socioeconomic conditions.* J Gerontol A Biol Sci Med Sci, 2005. **60**(2): p. 224-31.
32. Hamer, M., et al., *Feasibility of Measuring Sedentary Time Using Data From a Thigh-Worn Accelerometer.* Am J Epidemiol, 2020. **189**(9): p. 963-971.
33. Organization, W.H., *WHO technical specifications for automated non-invasive blood pressure measuring devices with cuff*. 2020, World Health Organization: Geneva.
34. Miller, M.R., et al., *Standardisation of spirometry.* Eur Respir J, 2005. **26**(2): p. 319-38.
35. Harwood, D.T. and D.J. Handelsman, *Development and validation of a sensitive liquid chromatography-tandem mass spectrometry assay to simultaneously measure androgens and estrogens in serum without derivatization.* Clin Chim Acta, 2009. **409**(1-2): p. 78-84.
36. Handelsman, D.J., et al., *Ultrasensitive Serum Estradiol Measurement by Liquid Chromatography-Mass Spectrometry in Postmenopausal Women and Mice.* J Endocr Soc, 2020. **4**(9): p. bvaa086.

For peer review only - http://bmjopen.bmj.com/site/about/guidelines.xhtml

37. Bohannon, R.W., et al., *Handgrip Strength: A Comparison of Values Obtained From the NHANES and NIH Toolbox Studies.* Am J Occup Ther, 2019. **73**(2): p. 7302205080p1-7302205080p9.
38. Statistics, A.B.o., *National Health Survey: First Results, 2017-18*, A.B.o. Statistics, Editor. 2018, Australian Bureau of Statistics: Canberra.
39. Mitchell, G.F., et al., *Hemodynamic correlates of blood pressure across the adult age spectrum: noninvasive evaluation in the Framingham Heart Study.* Circulation, 2010. **122**(14): p. 1379-86.
40. Shelley, J.M., et al., *Relationship of endogenous sex hormones to lipids and blood pressure in mid-aged women.* Ann Epidemiol, 1998. **8**(1): p. 39-45.
41. Szoeke, C., et al., *Predictive Factors for Verbal Memory Performance Over Decades of Aging: Data from the Women's Healthy Ageing Project.* Am J Geriatr Psychiatry, 2016. **24**(10): p. 857-67.
42. Koskova, I., et al., *Weight, body composition and fat distribution changes of Czech women in the different reproductive phases: a longitudinal study.* Prague Med Rep, 2007. **108**(3): p. 226-42.
43. Kuk, J.L., P.M. Janiszewski, and R. Ross, *Body mass index and hip and thigh circumferences are negatively associated with visceral adipose tissue after control for waist circumference.* Am J Clin Nutr, 2007. **85**(6): p. 1540-4.
44. Soriguer, F., et al., *Type 2 diabetes mellitus and other cardiovascular risk factors are no more common during menopause: longitudinal study.* Menopause, 2009. **16**(4): p. 817-21.
45. Rossetti, H.C., et al., *Normative data for the Montreal Cognitive Assessment (MoCA) in a population-based sample.* Neurology, 2011. **77**(13): p. 1272-5.
46. Santangelo, G., et al., *Normative data for the Montreal Cognitive Assessment in an Italian population sample.* Neurol Sci, 2015. **36**(4): p. 585-91.

FIGURE LEGENDS

Figure 1 The participant recruitment flow chart for the M-PreM study.

Figure 2 Timeline and number of participants for the existing surveys (S1-9) and the M-PreM data collection of the ALSWH 1973-78 cohort. The future surveys for this cohort are shown in grey. The M-PreM study provides baseline (wave 1, W1) data for a potential follow-up (W2) of the participants.

For peer review only

For peer review only - http://bmjopen.bmj.com/site/about/guidelines.xhtml

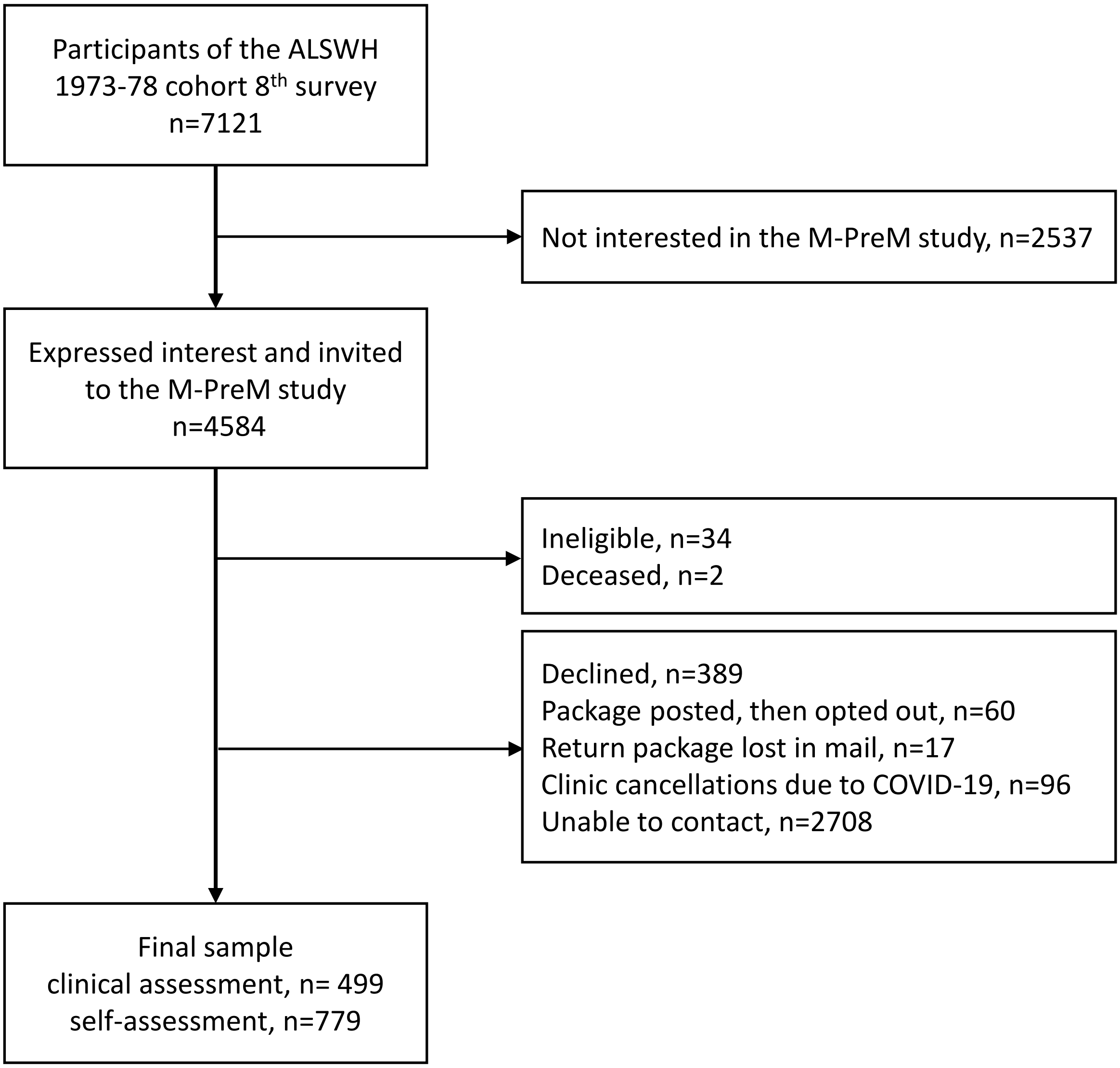
Participants of the ALSWH 1973-78 cohort 8th survey n=7121
Not interested in the M-PreM study, n=2537
Expressed interest and invited to the M-PreM study n=4584
Ineligible, n=34
Deceased, n=2
Declined, n=389
Package posted, then opted out, n=60
Return package lost in mail, n=17
Clinic cancellations due to COVID-19, n=96
Unable to contact, n=2708
Final sample
clinical assessment, n= 499
self-assessment, n=779

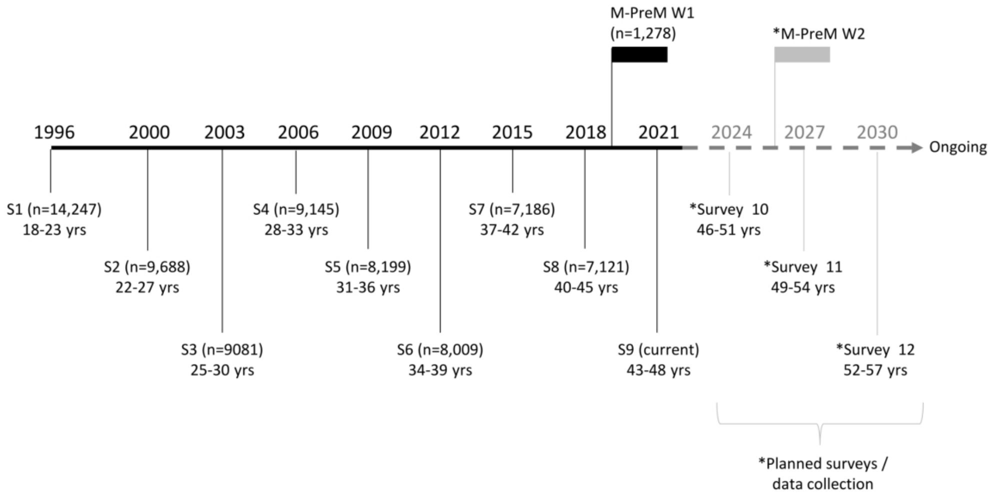

Figure 2 Timeline and number of participants for the existing surveys (S1-9) and the M-PreM data collection of the ALSWH 1973-78 cohort. The future surveys for this cohort are shown in grey. The M-PreM study provides baseline (wave 1, W1) data for a potential follow-up (W2) of the participants.

207x108mm (300 x 300 DPI)

STROBE Statement—Checklist of items that should be included in reports of ***cohort studies***

	Item No	Recommendation	Page No
**Title and abstract**	1	(*a*) Indicate the study's design with a commonly used term in the title or the abstract	1
		(*b*) Provide in the abstract an informative and balanced summary of what was done and what was found	3
**Introduction**			
Background/rationale	2	Explain the scientific background and rationale for the investigation being reported	4-5
Objectives	3	State specific objectives, including any prespecified hypotheses	6
**Methods**			
Study design	4	Present key elements of study design early in the paper	6
Setting	5	Describe the setting, locations, and relevant dates, including periods of recruitment, exposure, follow-up, and data collection	7-10
Participants	6	(*a*) Give the eligibility criteria, and the sources and methods of selection of participants. Describe methods of follow-up	6-7
		(*b*) For matched studies, give matching criteria and number of exposed and unexposed	n/a
Variables	7	Clearly define all outcomes, exposures, predictors, potential confounders, and effect modifiers. Give diagnostic criteria, if applicable	7-13
Data sources/ measurement	8*	For each variable of interest, give sources of data and details of methods of assessment (measurement). Describe comparability of assessment methods if there is more than one group	7-13
Bias	9	Describe any efforts to address potential sources of bias	n/a
Study size	10	Explain how the study size was arrived at	6-7
Quantitative variables	11	Explain how quantitative variables were handled in the analyses. If applicable, describe which groupings were chosen and why	n/a
Statistical methods	12	(*a*) Describe all statistical methods, including those used to control for confounding	n/a
		(*b*) Describe any methods used to examine subgroups and interactions	n/a
		(*c*) Explain how missing data were addressed	n/a
		(*d*) If applicable, explain how loss to follow-up was addressed	n/a
		(*e*) Describe any sensitivity analyses	n/a
**Results**			
Participants	13*	(a) Report numbers of individuals at each stage of study—eg numbers potentially eligible, examined for eligibility, confirmed eligible, included in the study, completing follow-up, and analysed	6-7
		(b) Give reasons for non-participation at each stage	6-7
		(c) Consider use of a flow diagram	6-7
Descriptive data	14*	(a) Give characteristics of study participants (eg demographic, clinical, social) and information on exposures and potential confounders	14-15
		(b) Indicate number of participants with missing data for each variable of interest	14-15
		(c) Summarise follow-up time (eg, average and total amount)	n/a
Outcome data	15*	Report numbers of outcome events or summary measures over time	16-17

For peer review only

Main results	16	(*a*) Give unadjusted estimates and, if applicable, confounder-adjusted estimates and their precision (eg, 95% confidence interval). Make clear which confounders were adjusted for and why they were included	n/a
		(*b*) Report category boundaries when continuous variables were categorized	16-17
		(*c*) If relevant, consider translating estimates of relative risk into absolute risk for a meaningful time period	n/a
Other analyses	17	Report other analyses done—eg analyses of subgroups and interactions, and sensitivity analyses	n/a
**Discussion**			
Key results	18	Summarise key results with reference to study objectives	n/a
Limitations	19	Discuss limitations of the study, taking into account sources of potential bias or imprecision. Discuss both direction and magnitude of any potential bias	17-18
Interpretation	20	Give a cautious overall interpretation of results considering objectives, limitations, multiplicity of analyses, results from similar studies, and other relevant evidence	n/a
Generalisability	21	Discuss the generalisability (external validity) of the study results	n/a
**Other information**			
Funding	22	Give the source of funding and the role of the funders for the present study and, if applicable, for the original study on which the present article is based	18

*Give information separately for exposed and unexposed groups.

Note: An Explanation and Elaboration article discusses each checklist item and gives methodological background and published examples of transparent reporting. The STROBE checklist is best used in conjunction with this article (freely available on the Web sites of PLoS Medicine at http://www.plosmedicine.org/, Annals of Internal Medicine at http://www.annals.org/, and Epidemiology at http://www.epidem.com/). Information on the STROBE Initiative is available at http://www.strobe-statement.org.

For peer review only

For peer review only - http://bmjopen.bmj.com/site/about/guidelines.xhtml
